# Chiral Induction and Memory via Supramolecular Deracemization

**DOI:** 10.1002/anie.202510584

**Published:** 2025-08-05

**Authors:** Robert Hein, Eric Sidler, Yohan Gisbert, Ben L. Feringa

**Affiliations:** ^1^ Stratingh Institute for Chemistry University of Groningen Nijenborgh 3 Groningen 9747 AG The Netherlands; ^2^ Organic Chemistry Institute University of Münster Corrensstraße 36 48149 Münster Germany

**Keywords:** Chiral induction, Chiral memory, Deracemization, Host‐guest systems, Molecular switch, Overcrowded alkene

## Abstract

Chirality is intrinsic to nature, and control of molecular chirality remains at the forefront of the chemical sciences. The introduction of fundamentally new principles to break symmetry, leading to the formation of a specific enantiomer, is particularly challenging with important implications in numerous scientific fields. Herein we present a helically chiral dynamic molecular switch whose enantiomers are connected via a single symmetric metastable state that can be populated via light or redox stimuli. Relaxation of the prochiral state in the presence of a bound chiral ammonium guest results in the preferential formation of a specific enantiomer through a conceptually unique supramolecular deracemization process. Importantly, the formed host enantiomer is stable even in the absence of guest, enabling reversible and stable chiral information transfer, while oxidation of the host results in an enantioenriched dication that is insensitive to light, allowing orthogonal chiral memory.

## Graphical abstract

The helical chirality of a dynamic crown‐ether switch can, by light‐ or redox‐switching to a prochiral state, be erased and subsequently stereoselectively re‐formed by relaxation in the presence of a point‐chiral ammonium guest. This unique supramolecular deracemization enables facile transfer and stable memory of chiral information.

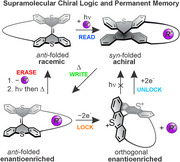

## Introduction

Controlling the formation of a distinct chiral state, in particular specific enantiomers of a molecule or supramolecular assemblies, is of utmost importance for numerous applications ranging from pharmaceuticals to catalysis and display materials.^[^ ^1^ ^]^ Fundamental questions on homochirality in relation to the origin of life,^[^ ^2^, ^3^ ^]^ chiral‐induced spin selectivity (CISS) effects,^[^ ^4^ ^]^ circularly polarized luminescence,^[^ ^5^, ^6^ ^]^ as well as chiral amplification^[^ ^7^, ^8^ ^]^ and chiral memory, among others, continue to stimulate the fascination of scientists.^[^ ^9^ ^]^ To this end, unconventional methods for desymmetrization and the transfer of chiral information between different molecular entities and across different length‐scales, remain particularly important but challenging.^[^ ^10^, ^11^ ^]^ In order to break the symmetry of a racemic state, “imprinting” the chiral information of a small‐molecule chiral “effector” onto a suitable substrate via intermolecular allosteric interactions is perhaps the most straightforward and appealing approach and obviates the (re)formation of covalent bonds.^[^ ^12^, ^13^ ^]^ This chiral induction strategy is especially powerful in the context of larger assemblies,^[^ ^14^, ^15^, ^16^ ^]^ in particular in (supramolecular) polymers,^[^ ^9^, ^17^, ^18^ ^]^ where even small amounts of chiral dopant can effectively control the formation of well‐defined chiral superstructures.^[^ ^19^ ^]^

**Figure 1:**
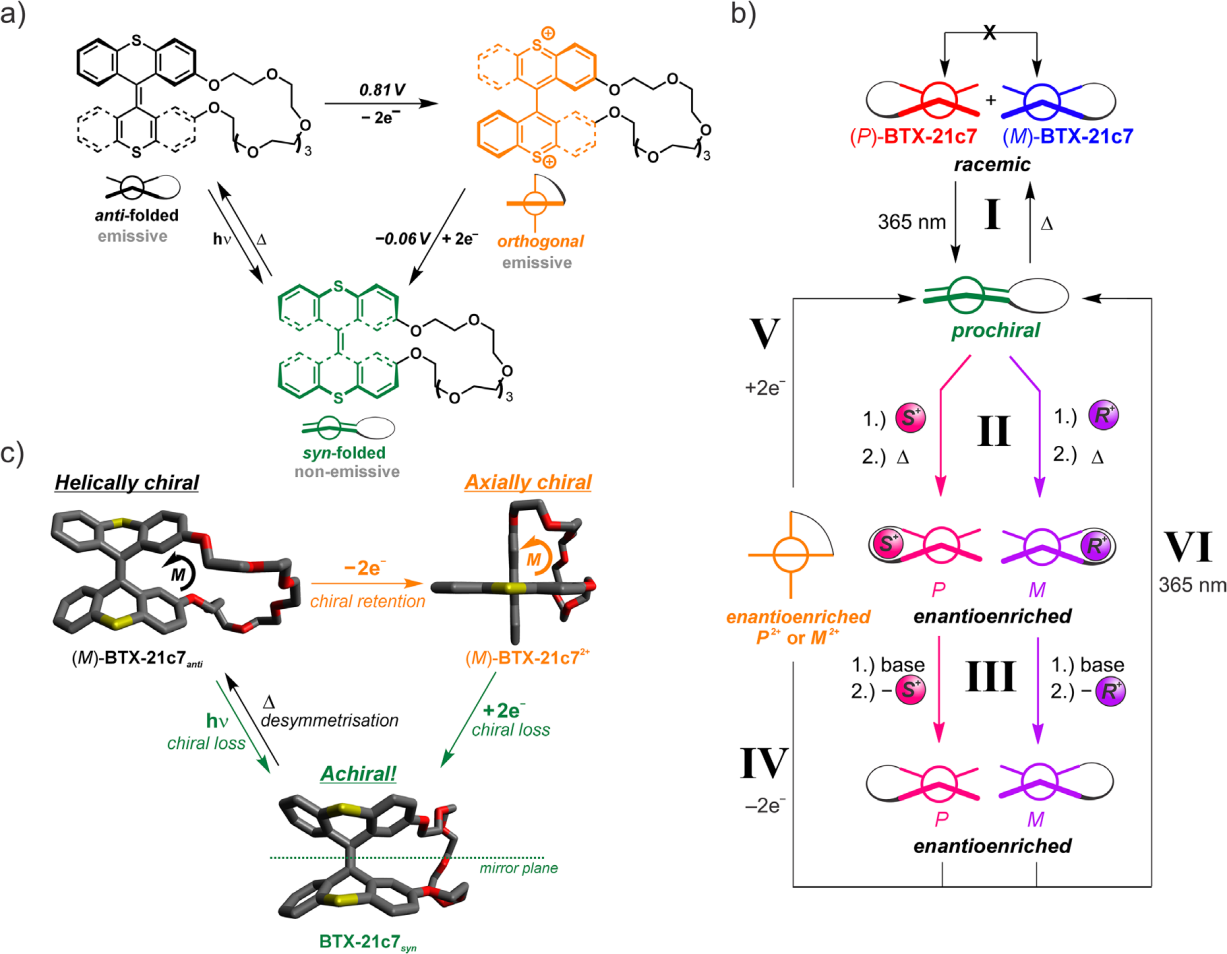
Chirality and induction thereof in **BTX‐21c7**. a) General switching scheme of BTX‐crown ethers.^[^ ^40^ ^]^ b) Schematic representation of the supramolecular deracemization concept of (*P*)/(*M*)‐**BTX‐21c7** and the possible interconversions (steps I‐VI) between all (chiral) states. In the most common iteration of the induction process, steps I and II are carried out subsequently in one pot, i.e., the chiral guest is already present during the irradiation step I. The chemical structures of the chiral ammonium guests (here depicted as colored balls) are displayed in Figure 2a. c) Changes in chirality upon switching as illustrated for DFT‐calculated structures of the lowest energy conformers of **BTX‐21c7** (see SI for further details). Hydrogen atoms are omitted for clarity.

We envisioned a fundamentally distinct principle combining a small‐molecule chiral guest with a transient, metastable, and symmetric prochiral host state to break the symmetry of a racemic mixture (Figure 1). The transfer of chiral information between a single host and chiral guest in a small, non‐covalently bonded complex has been used in numerous potential applications, e.g., chiroptical switches,^[^ ^20^ ^]^ enantioselective recognition,^[^ ^21^ ^]^ catalysis,^[^ ^22^ ^]^ sensing,^[^ ^23^, ^24^ ^]^ and information processing.^[^ ^13^, ^25^, ^26^ ^]^ An ultimate goal in this context is the permanent transfer and memory of chiral information at the single‐molecule level, even in the absence of the initial chiral inductor and in the absence of other stabilizing interactions such as in assemblies or aggregates.^[^ ^27^ ^]^ However, this has, to the best of our knowledge, remained elusive. Specifically, while various host‐guest systems in which the chirality of a host can be controlled by binding of a chiral guest are known,^[^ ^28^ ^]^ the induced host chirality is lost upon removal of the chiral guest,^[^ ^29^ ^]^ unless the host chirality is kinetically trapped^[^ ^30^ ^]^ or (permanently) locked by a secondary process^[^ ^31^ ^]^ such as protonation,^[^ ^32^ ^]^ sub‐component exchange,^[^ ^29^, ^33^, ^34^ ^]^ redox,^[^ ^35^ ^]^ or covalent reactions.^[^ ^36^, ^37^ ^]^ Alternatively, an asymmetric environment such as a chiral solvent can also be used to induce (permanent) chirality in a photo‐switchable system,^[^ ^38^ ^]^ however, this relies on a vast excess of the inducer and is not mediated by specific host‐guest interactions.

Herein we present a distinctive and simple methodology for the non‐covalent transfer and permanent memory of chirality in a discrete two‐component host‐guest system. This was achieved by using a dynamic crown ether receptor based on a bisthioxanthylidene (BTX) molecular switch, which can, in response to either light or redox stimulation, be switched between three distinct molecular states that possess different geometries, polarities, and optical properties, including different colors and fluorescence (Figure 1a).^[^ ^39^ ^]^

Key to our approach is the conversion of the racemic *rac*‐**BTX‐21c7** to a metastable and symmetric prochiral *syn*‐folded state which allows desymmetrization by the chiral guest to bias the formation of enantioenriched (*P*)‐ or (*M*)‐**BTX‐21c7** (Figure 1b). The transformation to the transient prochiral *syn*‐folded state can be triggered by redox or light and enables a unique chiral induction mechanism (Figure 1b,c).

In the molecular design of the **BTX‐21c7** receptor, we take advantage of the large, switchable molecular changes that can be efficiently transduced to modulate its cation binding properties.^[^ ^40^ ^]^ Specifically, for the two neutral states of **BTX‐21c7**, the lowest energy *anti*‐folded state, as well as the metastable *syn‐*folded state, typically display significant cation binding affinities. In contrast, the dicationic, orthogonal state expectedly does not interact with cations. As shown in Figure 1a, each of these states can be quantitatively populated and interconverted using either light/heat or redox. More precisely, the orthogonal state can be accessed by two‐electron oxidation of the neutral (*anti*‐folded) state, and the metastable *syn*‐folded state can either be obtained by reduction of the dication or by UV–light irradiation of the *anti*‐folded state.

**Figure 2:**
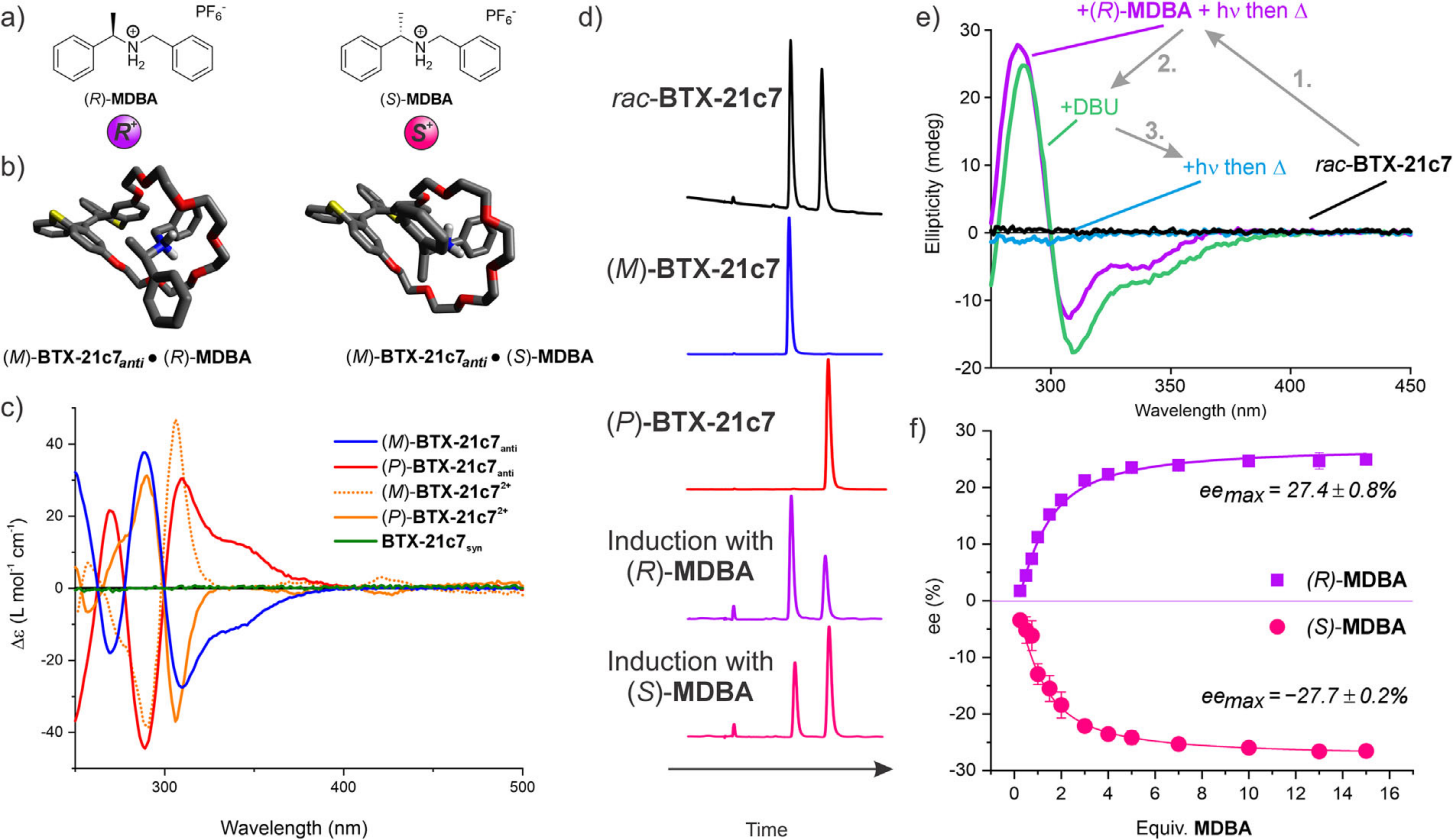
Chiral induction with **MDBA**. a) Chemical structures of chiral ammonium guests used herein. b) DFT‐calculated structures of the host‐guest complexes of (*M*)‐**BTX‐21c7** *_**anti**_* with (*R*)‐ or (*S*)‐**MDBA** showing the pseudorotaxane structure of these diastereomeric complexes. Most hydrogen atoms are omitted for clarity. Calculations were performed on ensembles of conformers, following a workflow described in the Supporting Information. The final geometry optimization of the lowest‐energy conformers displayed here was performed at the r^2^SCAN‐3c/SMD(CH_2_Cl_2_) level of theory.^[^ ^44^, ^45^ ^]^ c) CD spectra of all five geometric/switching states of **BTX‐21c7** (∼50 µM in CH_2_Cl_2_). The corresponding UV–vis spectra are shown in Figures S24 and S28. d) HPLC chromatograms of pure *rac‐*, (*M*)‐ and (*P*)‐**BTX‐21c7** *_**anti**_*, as well as after light‐triggered induction in the presence of (*R*)‐ or (*S*)‐**MDBA**. Note that the **MDBA** guest does not co‐elute with the host, but is chromatographically separated (Figure S12). e) Changes in CD spectra at different steps of the chiral induction process. The small initial peak at lower retention time is the injection peak, which is comparatively more visible in the two lower panels due to a lower concentration of the switch. f) Dependence of enantiomeric excess (*ee*) of (*M/P*)‐**BTX‐21c7** *_**anti**_* (250 µM in CH_2_Cl_2_) on the concentration of (*S*)*‐ or* (*R*)‐**MDBA** after light‐triggered induction. Error bars represent one standard deviation of independent triplicate repeats.

During our ongoing investigations into overcrowded alkene switching systems,^[^ ^41^, ^42^ ^]^ we realized that the BTX scaffold also displays rich chiral properties.^[^ ^43^ ^]^ Both the *anti*‐folded as well as orthogonal switching states are helically or axially chiral and thus possess two stable *P*/*M* enantiomers, which do not interconvert at room temperature (Figures 1c and 2c). The *syn*‐folded state, however, is C_S_ symmetric and contains a mirror plane perpendicular to the central double bond. Importantly, this means that any chiral information “stored” in the *anti*‐folded or orthogonal states is completely lost when the system is switched to the *syn*‐folded state (Figure 1c). In turn, the *syn*‐folded state acts as a prochiral precursor, which thermally relaxes to a racemic mixture of (*M*)‐ and (*P*)‐**BTX‐21c7*_anti_***.

## Results and Discussion

### Chiral Induction via Host‐Guest Complexation

The relaxation pathway of the *syn*‐folded state can indeed be biased towards one helical enantiomer by binding of a chiral guest (Figure 1b). Upon relaxation of the **BTX‐21c7** receptor from its *syn‐*folded state in the presence of the enantiopure point‐chiral methyl dibenzyl ammonium (**MDBA**) guest, which forms pseudo‐rotaxanes with the host (Figure 2a, b), significant chiral induction was observed by circular dichroism (CD) spectroscopy and high‐performance liquid chromatography (HPLC, Figure 2d). This process is operationally simple and fast, as the *syn‐*folded state can easily be generated quantitatively within seconds by UV‐light irradiation of the native *anti‐*folded receptor while thermal back‐relaxation is also completed within ∼3 min at room temperature. Crucially the dynamic nature of this switching process, combined with the non‐covalent and reversible binding of the chiral ammonium guest enables facile and reconfigurable chiral memory.

Specifically, the helical inversion barrier of **BTX‐21c7*_anti_*** is with ∼111.1 kJ mol^−1^ sufficiently large to prevent racemization at room temperature (t_1/2, 20 °C _= 82 d, Figures S21 and S22), also enabling separation of (*P*)‐**BTX‐21c7*_anti_*** and (*M*)‐**BTX‐21c7*_anti_*** by chiral HPLC (Figure 2d).^[^ ^46^ ^]^ The absolute configuration of these enantiomers was determined by DFT calculations as detailed in the Section S6. Following irradiation of (*P*)‐**BTX‐21c7*_anti_*** with 365 nm light, full racemization was observed (Figure 1b, step I followed by thermal relaxation, and Figure S7). However, irradiation, and subsequent thermal relaxation of **BTX‐21c7*_anti_*** in CH_2_Cl_2_ in the presence of (*S*)‐**MDBA** or (*R*)‐**MDBA** induces preferential formation of (*P*)‐**BTX‐21c7*_anti_*** or (*M*)‐**BTX‐21c7*_anti_***, respectively, with an enantiomeric ratio (*er*) of 64:36 (Figures 1b and 2d, step I+II). The outcome of this induction is independent of the initial enantiomeric ratio of the host as the observed *er* is the same regardless if starting from (*P*)‐ or *rac‐* **BTX‐21c7*_anti_*** (Figures 2d, S5, and S8). At the same time, performing the chiral induction of (*P*)‐**BTX‐21c7** with either guest enantiomer leads to an opposite enantiomer of the host (Figures S8 and S9), confirming that in all cases the same prochiral *syn‐*folded state is quantitatively populated. Importantly, this enables not only deracemization of the racemic host but also controlled “overwriting/erasing” of chiral information from one enantiomer to the other (vide infra).

As expected for a non‐covalent chiral induction process, the degree of enantio‐induction is dependent on the concentration of **MDBA**, with a plateau from ∼10 equiv. of guest (Figure 2f). The crucial role of host‐guest complexation was further confirmed by control experiments using the free‐base, non‐binding form of the chiral guest, whereby no induction was observed (Figure S6). Similarly, the guest in principle only needs to be present during the relaxation step (step II, Figure 1b); light‐induced generation of **BTX‐21c7*_syn_***, followed by subsequent addition of (*S*/*R*)‐**MDBA** and thermal relaxation afforded the same *er* as observed when the chiral guest is already present during irradiation (Figure S10); i.e., irradiation is only required to populate the transient prochiral *syn‐*folded state. This confirms that induction occurs in the electronic ground‐state, i.e., is a purely thermal process and is not related to excited‐state phenomena. In other words, light is simply a convenient stimulus to easily, quantitatively generate the prochiral *syn‐*folded state. As a result, the degree of induction is also not dependent on the number of irradiation cycles (Figure S26).

Crucially, removal of the ammonium guest after induction *does not* induce loss of chiral information; the chirality is effectively locked in the *anti*‐folded state (Figure 1b, step III). This was confirmed by both HPLC and CD spectroscopy, whereupon deprotonation, and hence decomplexation of the **MDBA** guest, no change in *er* was observed (Figure 2e). Racemization occurred only after subsequent irradiation to form the *syn*‐folded state. These observations crucially highlight the unique utility of the system for reversible chiral memory and information storage. To the best of our knowledge, this represents the first example of a discrete two‐component non‐covalent host‐guest system in which no aggregation, assembly, or follow‐up process is required to permanently maintain the induced state. Specifically, it represents a chiral supramolecular memory system^[^ ^15^ ^]^ in which the kinetic stability is strongly dependent on the conformational state; i.e., very high kinetic inertness to racemization is observed in the relaxed, lowest energy *anti‐*folded state. In contrast, in other discrete supramolecular chiral memory systems, induction by guest binding is commonly carried out in a labile lowest energy state and then locked via specifically designed subsequent, and sometimes irreversible, processes.^[^ ^29^, ^32^, ^33^, ^34^, ^35^, ^36^, ^37^ ^]^ Without such a follow‐up process, the induced host chirality would otherwise be erased upon guest removal, or can only be maintained at temperatures that are significantly lower than those at which the initial induction was carried out.^[^ ^30^ ^]^

Here, the volatility of the induced state in the absence of a guest is obviated by the unique interplay of chiral and switching properties; only the *syn‐*folded state is configurationally labile (prochiral). A distinctive feature is that its generation, and subsequent relaxation are required to achieve induction or racemization.

### Mechanistic Studies of Deracemization

**Figure 3:**
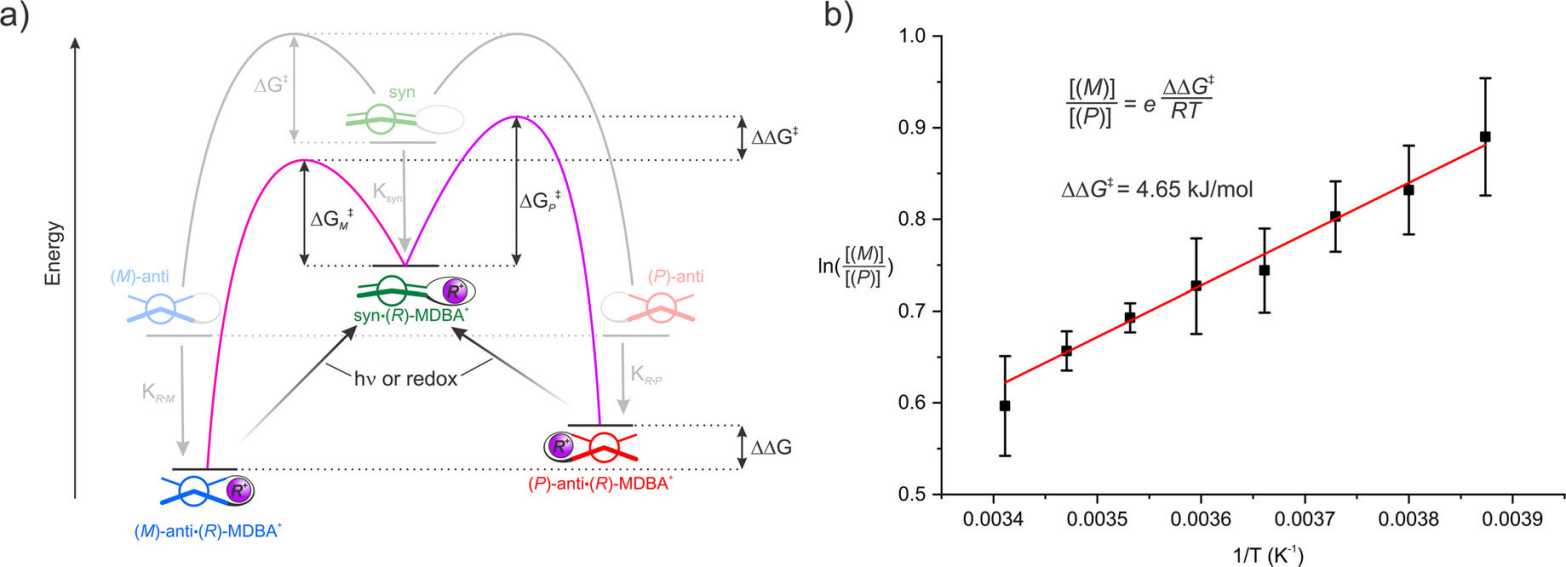
Energetic considerations for deracemization of **BTX‐21c7**. a) Schematic energy diagram for the supramolecular deracemization of **BTX‐21c7**, which is akin to a (dynamic) kinetic resolution. The relative energies are not drawn to scale. b) Linear relationship of the natural logarithm of the enantiomeric ratio and the inverse temperature (from −15 °C to +20 °C), according to a Curtin‐Hammett‐type system. Error bars represent one standard deviation of independent triplicate repeats.

This was further confirmed by induction attempts using only *rac‐* **BTX‐21c7*_anti_*** and (*R*)‐**MDBA** without switching to the *syn*‐folded state, where no induction was observed at room temperature (Figure S4). Even upon heating to 80 °C in 1,2‐dichloroethane (DCE) for a prolonged time, at which (*P*)‐**BTX‐21c7*_anti_*** and (*M*)‐**BTX‐21c7*_anti_*** (and their MDBA complexes) can slowly interconvert, an *er* of only 57:43 was achieved (Figure S20). This value directly reflects the difference in binding energy between the (*R*)‐**MDBA**•(*M*)‐**BTX‐21c7*_anti_*** and (*R*)‐**MDBA**•(*P*)‐**BTX‐21c7*_anti_*** complexes (ΔΔG = ΔG_*R*•*M*_ − ΔG_*R*•*P*_ = −0.83 kJ mol^−1^ at 80 °C), see also Figure 3a. As these complexes are diastereomers one would expect subtle differences in their structures and thermodynamic stabilities, as also confirmed by DFT calculations (Figure 2b and Section S6). The energy difference ΔΔG was also experimentally assessed by ^1^H NMR titrations of (*M*)‐**BTX‐21c7*_anti_*** with (*R*)‐**MDBA** or (*S*)‐**MDBA** (Figures S13–S15) which revealed stronger binding to the former guest enantiomer with $K_{M•R}=26900\pm 6900\;M^{-1}$ and $K_{M•S}=18700\pm 2300\;M^{-1}$ at room temperature, respectively. This corresponds to a ΔΔG of −0.89 kJ mol^−1^ (i.e., an *er* of 59:41), which is very similar to the value determined by induction under thermodynamic control. As discussed above, the asymmetric induction of *rac‐* **BTX‐21c7** using (*R*)‐**MDBA** does indeed lead to formation of (*M*)‐**BTX‐21c7*_anti_*** (Figure 2d), i.e., the helically chiral enantiomer of the host which preferentially binds to (*R*)‐**MDBA**. The chiral induction can thus be carried out by heating, which leads to generation of the same host‐enantiomer. However, light‐triggered induction, and subsequent relaxation of **BTX‐21c7*_syn_*** is not only operationally much faster, as the half‐life time of the *syn*‐folded state in the presence of the chiral guest is only ∼30 s at room temperature (Figures S18 and S19), but also proceeds with a significantly larger *er* (64:36 at r.t.). Hence, the ground state energy difference ΔΔG cannot be the (sole) determinant for the whole degree of induction observed, but only qualitatively indicates which enantiomer is preferably formed. This indicates that the degree of stereoselection is primarily driven by differences in the transition state (TS) energies of the divergent pathways (ΔΔG^‡^, Figure 3a), i.e., a kinetic effect. Indeed, slowing down the thermal relaxation by lowering the temperature resulted in an increase in *er* (72:28) at −15 °C. A further decrease in temperature would result in even larger *er*, however, at the cost of a slowed‐down process (at −15 °C full relaxation took ∼130 min). Performing the asymmetric induction at varying temperatures under kinetic control (−15 °C to 20 °C) enabled determination of the difference in TS energies of ΔΔG^‡^ = 4.65 kJ mol^−1^, by applying principles of a Curtin–Hammett‐type system (Figure 3b).^[^ ^47^, ^48^ ^]^

This process is hence conceptually related to dynamic kinetic resolution (DKR) but fundamentally differs from classic DKR in that there is only one (prochiral) high‐energy state. More generally, the concept described herein corresponds to a deracemization, a highly desired atom‐economic approach to access enantioenriched compounds.^[^ ^49^, ^50^, ^51^ ^]^ However, the vast majority of the reported deracemizations occur via reactions involving the (re)formation of covalent bonds and typically require multiple reagents, highly controlled reaction conditions, and/or (photo)catalysts.^[^ ^52^, ^53^, ^54^, ^55^ ^]^ In contrast to DKR or established deracemizations, the supramolecular deracemization principle presented here proceeds without any covalent modifications/reactions, is operationally simple, fast, and furthermore requires no additives apart from a simple chiral ammonium salt as guest. Specifically, in the standard, most operationally simple implementation, the host and a small excess of the enantiopure point‐chiral guest are simply mixed in CH_2_Cl_2_ and then briefly irradiated with UV light. Depending on the concentration of **BTX‐21c7** and intensity of the light source, quantitative switching to the *syn‐*folded state can be achieved within a few tens of seconds and can be conveniently monitored by the naked eye as the initial blue fluorescence of the *anti‐*folded state vanishes upon complete photo‐switching. This photoswitching process is quantitative (Figure S16), because irradiation with light only promotes the *anti* → *syn* conversion, while the reverse reaction is a purely thermal process.^[^ ^56^ ^]^ Following the irradiation, full thermal relaxation and the concomitant deracemization then proceed within ∼3 min, such that a whole induction experiment can be carried out in less than 5 min under ambient conditions. Note that the outcome of the induction is the same irrespective of how the prochiral *syn‐*folded state is generated (either via photoswitching from the *anti‐*folded state or via reduction of the dicationic state) nor whether the **MDBA** guest is present during the switching to the *syn‐*folded state or added after the switching.

### Orthogonal Chiral Information Transfer and Storage

**Figure 4:**
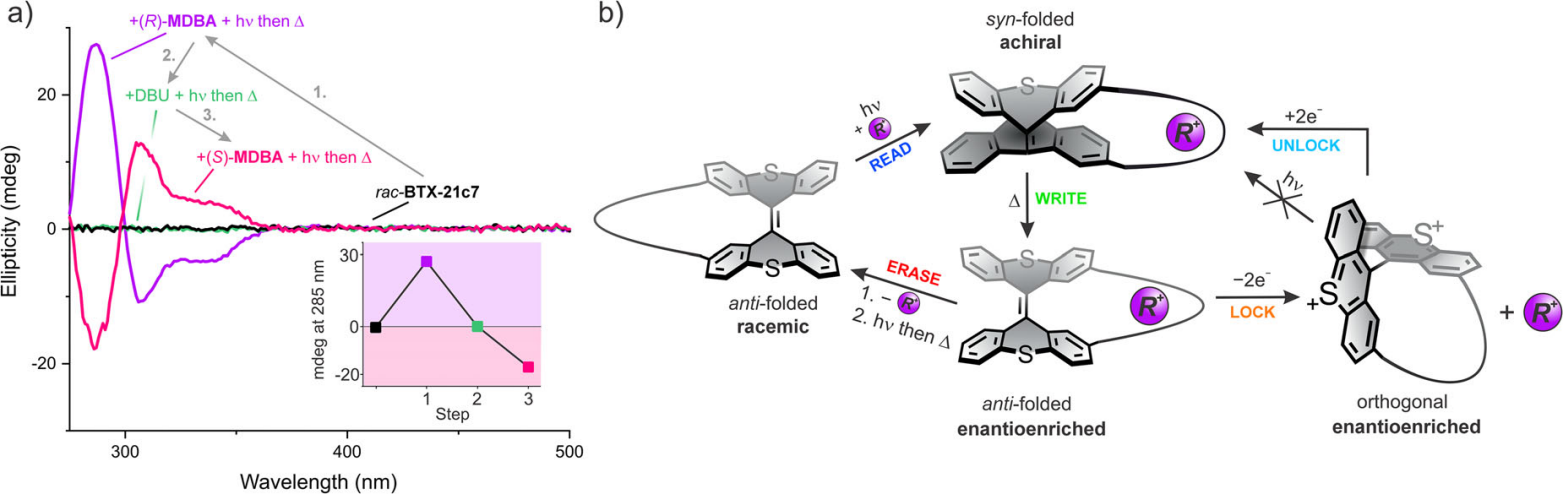
Chiral information storage. a) Changes in CD spectra of 50 µM **BTX‐21c7** in CH_2_Cl_2_ upon subsequent chiral induction with (*R*)‐**MDBA** (8 equiv., step 1 “writing”), racemization with DBU and light (8 equiv., step 2, “erasing”), and induction with (*S*)‐**MDBA** (80 equiv., step 3, “rewriting”), all in one pot. The inset displays the CD changes observed at 285 nm for the individual steps. b) Schematic depiction of the chiral information transfer between the dynamic switch and the chiral information source (here chiral guest, R^+^) whose chirality can be “read” by the prochiral *syn*‐folded state and upon relaxation to the *anti‐*folded state “written” into the helicity of the switch. Upon removal of the guest and an irradiation/relaxation cycle, full racemization leads to “erasing” of the chiral information. Alternatively, oxidation of the enantioenriched *anti‐*folded state leads to a “locking” of the system; the dication is both configurationally stable and unresponsive to light.

Importantly, our findings enable the non‐covalent transfer and, crucially, reversible storage of chiral information, as the chirality of the formed products ((*M/P*)‐**BTX‐21c7*_anti_***) is configurationally stable, yet easily reconfigurable (Figure 4).

For example, starting from *rac‐* **BTX‐21c7*_anti_***, chiral information can first be written and stored in the form of the (*M*)‐**BTX‐21c7*_anti_*** state upon induction with (*R*)‐**MDBA**. This state can then be maintained and non‐destructively assessed (e.g., via CD), either in the presence or absence of the initial chiral inductor (Figure 4a, step 1). Upon removal of the guest (e.g., via deprotonation or separation), the chirality of the system can easily be erased again via irradiation and relaxation (Figure 4a, step 2 and Figure 1b, step VI and I). Alternatively, the opposite chiral information can be overwritten and stored in the system by subsequent induction with (*S*)‐**MDBA**, resulting in the (*P*)‐**BTX‐21c7*_anti_*** state (Figure 4a, step 3).

Finally, it should be noted that the *syn*‐folded state can also be quantitatively populated via the reduction of the dication (Figure 1b, step V and Figure 4b), enabling chiral induction via redox stimuli. Indeed, chemical reduction of the isolated *rac‐* **BTX‐21c7^2+^** in the presence of (*R*)‐**MDBA** leads to the preferential formation of (*M*)‐**BTX‐21c7** with the same *er* as observed via light‐driven induction (Figure S11). Upon oxidation of the (enantioenriched) **BTX‐21c7*_anti_*** (Figure 1b, step IV), binding of the ammonium cation is completely turned off, thereby inducing ejection of the guest (Figure 4b).^[^ ^40^ ^]^ However, more importantly, the helical/axial chirality, and thus the chiral information, is retained and accompanied by significant changes in the chiroptical fingerprint of the system (see CD, Figure 2c). Additionally, the configurational stability of this non‐volatile dicationic state is even larger as no racemization was observed for more than 3 h at 80 °C (Figure S23). Furthermore, the dication is not light‐responsive such that oxidation removes this species from the dynamic cycle, thereby “locking” the chirality (Figure 4b). Only upon subsequent reduction can the species be deliberately “unlocked” and reintegrated into the cycle. These observations highlight the potential of this system for facile multistate chiral molecular logic and memory, which can be reversibly controlled under ambient conditions via orthogonal light and/or redox stimuli. Additionally, both light‐ and redox‐driven switching can be carried out with a very high degree of repeatability (Figures S24–S29, and Section S5).

## Conclusions

Reversible control and memory of the helical chirality of a dynamically switchable crown‐ether host **BTX‐21c7** was achieved by virtue of the non‐covalent transfer of chiral information from a point‐chiral ammonium guest via a conceptually novel supramolecular deracemization. Key to this strategy is the redox‐ or light‐driven formation of a metastable, prochiral *syn*‐folded receptor state, which by binding of one enantiomer of the chiral guest thermally relaxes to the *anti‐*folded conformation of the receptor with a preferred *P* or *M* helical chirality. While the thermodynamic stability (i.e. binding strength) of the diastereomeric host‐guest complexes determines how the point‐chirality of the guest regulates the helical chirality, the full extent of chiral enrichment can only be explained by considering the differences in transition state energies, i.e., by a type of DKR. Consequently, the degree of enantioenrichment can be judiciously controlled both by host/guest ratio as well as temperature, with enantiomeric ratios of up to 72:28.

Crucially, after the non‐covalent chiral induction, the resulting host chirality remains unaltered, even in the absence of the initial guest. To the best of our knowledge, this presents the first example of a chiral induction and stable memory process in a discrete, small‐molecule host‐guest system in which no specific follow‐up process is required to lock the host chirality. As a result this system is uniquely versatile, as demonstrated by multi‐step chiral information storage and processing. Specifically, we showed that receptor chirality can, at will, be switched between *P* or *M* or *rac* chiral states in a defined sequence in one pot under ambient conditions in an operationally simple and fast manner using light. Additionally, redox can be used as an orthogonal stimulus to convert the helical *anti‐*folded receptor to a highly stable, axially chiral dication **BTX‐21c7^2+^**, which is no longer light responsive and can thus be removed/added from/to the dynamic light‐driven switching cycle on demand.

We envision that these findings will enable numerous applications in molecular machines, chiral computing, logic and memory, and pave the way towards further fundamental studies of (supra)molecular chirality transfer.

## Conflict of Interests

The authors declare no conflict of interest.

## Supporting information



Supporting Information

Supporting Information

## Data Availability

The data that support the findings of this study are available in the Supporting Information of this article.
