# Supplementary material for: Chiral Induction and Memory via Supramolecular Deracemization
Source: Angew Chem Int Ed Engl. 2025 Aug 5;64(38):e202510584. doi: 10.1002/anie.202510584 (PMC12435425; doi:10.1002/anie.202510584)
Supplement: Supplementary file 1 — Supporting Information [file ANIE-64-e202510584-s002.pdf]

Supporting Information

Chiral Induction and Memory via Supramolecular Deracemization

Robert Hein,+,1,2 Eric Sidler,+,1 Yohan Gisbert1 and Ben L. Feringa*,1*

[+] Equal contributing first authors

*To whom correspondence should be addressed:

robert.hein@uni-muenster.de, b.l.feringa@rug.nl

[1] Stratingh Institute for Chemistry, University of Groningen, Nijenborgh 3, 9747 AG Groningen, The Netherlands

[2] Organic Chemistry Institute, University of Münster, Corrensstraße 36, 48149 Münster, Germany

Contents

1. Experimental Details 2

1.1 General Remarks 2

1.2 Synthesis 2

1.3 HPLC Measurements 4

1.4 CD Measurements 5

1.5 UV-Vis Measurements 5

1.6 ^{1}H NMR Irradiation/Titration Studies 6

1.7 Spectroelectrochemistry 6

1.8 Computational Methods 7

2. Chiral Induction Control Experiments 8

3. Mechanistic Studies 15

4. Determination of Racemization Barriers of BTX-21c7 and BTX-21c7^{2+} 22

5. Cycling Experiments 24

6. Calculated Host-Guest Structures and Energies 29

7. References 35

1. Experimental Details

1.1 General Remarks

All chemicals and solvents were obtained from commercial suppliers and used as received. Unless otherwise stated, all experiments were carried out using HPLC grade CH_2Cl_2.

Note that the nomenclature of the title compound **BTX-21c7** does not strictly follow the standard naming system of crown ether derivatives in that not all atoms of the overall macrocycle are counted herein. Specifically, we disregard the atoms of the BTX core in the fjord region, as this area is so sterically crowded that it does not contribute to the overall size of the macrocycle. The **BTX-21c7** crown ether is thus technically 6 atoms larger (*i.e.* BTX-27c7) but more closely resemble the parent, non-extended analogues (21-crown-7).

It should also be noted that in the *anti*-folded state the receptor possesses two helices with opposite helicity. The *P* and *M* chirality descriptors used herein always refer to the functionalised, *i.e.* crown-ether-containing, half of the title compound.

1.2 Synthesis

(S)-**MDBA** and *(R)*-**MDBA** were prepared according to an adapted literature procedure from their corresponding free base amines (>98% *ee*, obtained from Acros Organics).[1] Briefly, 1 g (4.73 mmol, 1 equiv.) of enantiopure *N*-benzyl-α-methylbenzylamin was suspended in 20 mL water under fast stirring. To this mixture, 0.92 mL of a 55 wt.% HPF_6 solution in water (5.73 mmol, 1.21 equiv.) was added dropwise. After 30 min, this solution was extracted with CH_2Cl_2 (2 x 50 mL) and the combined organic phases washed with an aqueous solution of NH_4PF_6 (0.1 M, 3 x 50 mL). The organic phase was then dried with anhydrous $MgSO_4$ and the residue thoroughly dried under vacuum. This afforded 0.94 g (2.63 mmol, 56%) of the product as white crystalline solid. The ^{1}H NMR spectrum of this compound is in good agreement with literature.[1-2]

The synthesis of **BTX-21c7** was carried out as recently described and the data was in accordance with literature.[3]

BTX-21c7^{2+}(ClO_4^-)$_2$

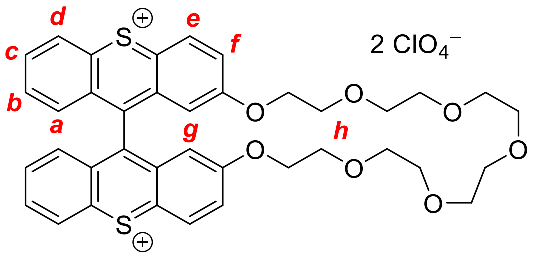

BTX-21c7 (3.3 mg, 0.005 mmol, 1 equiv.) was dissolved in 1.5 mL CH_2Cl_2 and 0.5 mL CH_3CN. To this was added a spatula of $Fe(ClO_4)_3$•xH_2O (excess) and the mixture was sonicated for 5 min. The organic phase was then washed with 3 x 1 mL water, dried over $MgSO_4$, filtered and concentrated in vacuo, affording 3.3 mg (77%) of **BTX-21c7^{2+}(ClO_4^-)$_2$** as a dark red/purple solid.

^{1}H NMR (300 MHz, CD_3CN) δ 9.08 – 8.93 (m, 4H), 8.33 (t, J = 7.8 Hz, 2H), 8.27 – 8.12 (m, 2H), 7.81 (t, J = 7.9 Hz, 2H), 7.56 (d, J = 8.9 Hz, 2H), 6.82 (d, J = 2.4 Hz, 2H), 3.96 – 3.77 (m, 4H), 3.72 – 3.61 (m, 2H), 3.54 – 3.34 (m, 18H).

^{13}C NMR (151 MHz, CD_3CN) δ 162.8, 157.2, 148.8, 146.6, 137.6, 133.7, 133.4, 132.8, 132.8, 131.1, 130.6, 129.3, 111.5, 71.3 (2x), 71.1, 71.1, 70.5, 69.3.

HR-ESI-MS (+): *m/z* calcd. for $C_{38}H_{38}NaO_7S_2^+$ $[M+Na]^+$: 693.1951, found: 693.1951.

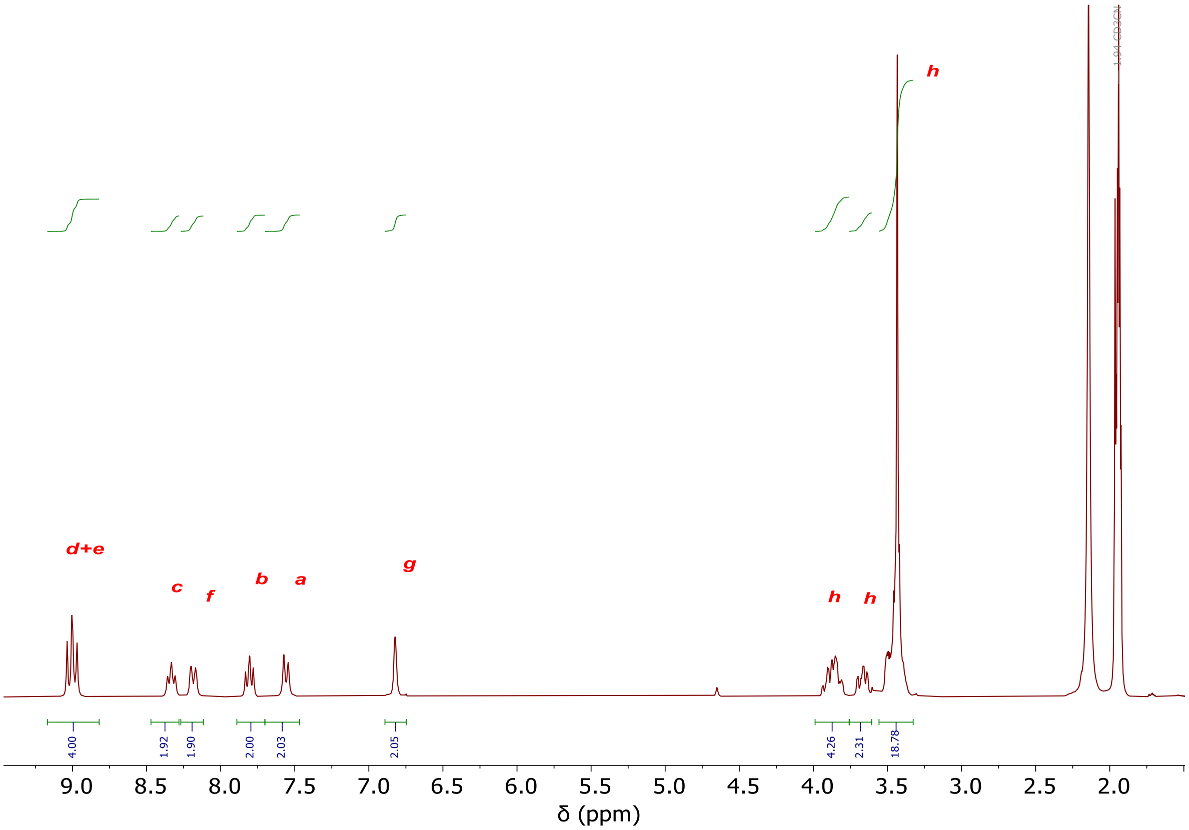

Figure S1. ^{1}H NMR spectrum of **BTX-21c7^{2+}(ClO_4^-)$_2$** (300 MHz, CD_3CN).

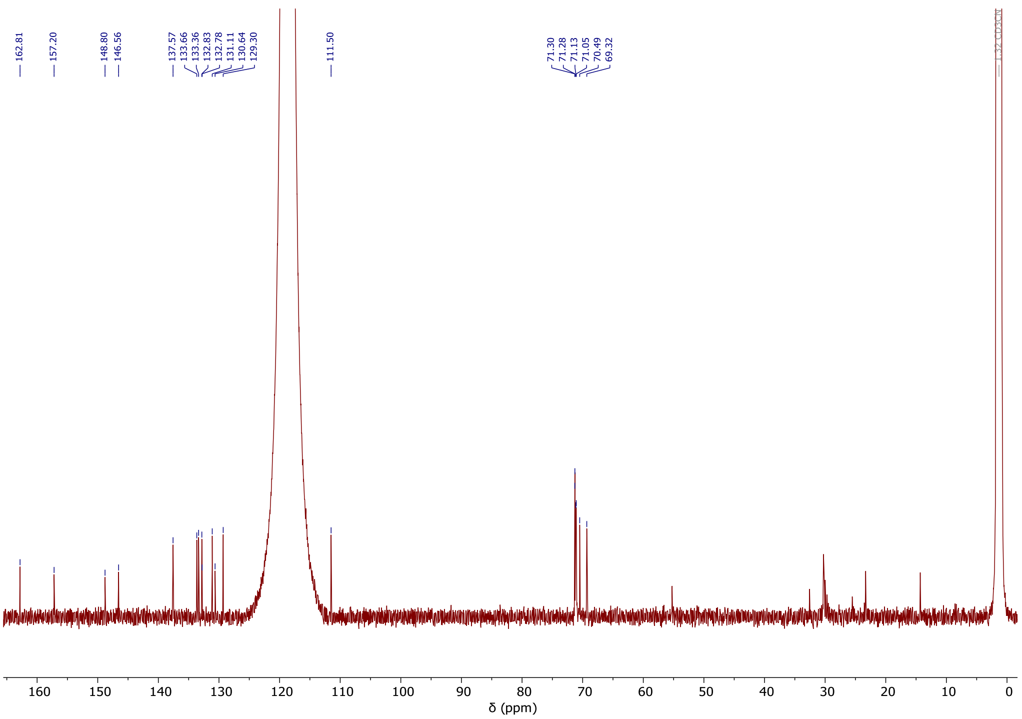

Figure S2. ^{13}C NMR spectrum of **BTX-21c7^{2+}(ClO_4^-)$_2$** (151 MHz, CD_3CN). The unmarked peaks in the aliphatic region are from residual grease.

1.3 HPLC Measurements

All HPLC measurements were performed using a Shimadzu Prominence HPLC system equipped with a CHIRALPAK IA column from Daicel Corporation and a photo diode array detector.

Chiral Resolution

Analytical separation of (*M*)-**BTX-21c7** and (*P*)-**BTX-21c7** was performed on a 4.6x250 mm column with a particle size of 5 µm, using a *n*-heptane/isopropanol (8:2) mixture as mobile phase and a flowrate of 1 mL/min at 40 °C column oven temperature. A sample of *rac*-**BTX-21c7** was injected as a solution in CH_2Cl_2.

Semipreparative separation was performed on a 10x250 mm column with a particle size of 5 µm, using a *n*-heptane/isopropanol (8:2) mixture as mobile phase and a flowrate of 5 mL/min at 40 °C column oven temperature. A sample of *rac*-**BTX-21c7** was injected as a solution in CH_2Cl_2.

Assignment of absolute configuration is based on TD-DFT calculations, see Section 5.

Chiral Induction Titration Studies

The chiral induction titration studies were performed in independent triplicates for both *rac*-**BTX-21c7** with (*R*)-**MDBA**, and *rac*-**BTX-21c7** with (*S*)-**MDBA**. Typically, 1.5 mL HPLC vials, containing a low-volume inset, were charged with 200 µL of a CH_2Cl_2 solution containing 250 µM *rac*-**BTX-21c7** and varying amounts of either (*R*)-**MDBA** or (*S*)-**MDBA** (0 to 15

equivalents). The samples were then irradiated with a broadband UV-A lamp (CHX-FL-A-100W, ~365 nm) for ~1-2 min before letting them relax at room temperature. Conveniently, the degree of photoswitching can easily be assessed by the naked eye, as the initial blue fluorescence of the *anti*-folded receptor state vanishes upon switching to the *syn*-folded state. An aliquot of 7 µL was then injected into the HPLC using the same conditions described in section 1.3. The enantiomeric ratio was determined by calculating the integral of both (*P*)-**BTX-21c7** and (*M*)-**BTX-21c7** in the chromatogram (PDA detector extract at 370 nm).

1.4 CD Measurements

CD measurements were carried using a Jasco J-715 or J-815 spectropolarimeter with a 1 cm pathlength quartz cuvette. For low temperature studies a CoolSpek holder was used. If required, irradiation was carried out using a Thorlabs LED (M365) at a 90° angle in a stirred cuvette.

Low-Temperature Induction Studies

A solution containing 50 µM *rac*-**BTX-21c7** and excess (*R*)-**MDBA** (3.7 mM) in CH_2Cl_2 was placed in a quartz cuvette equipped with a stirring bar and cooled to a specific temperature (between -15 °C and 20 °C) in the CD spectrometer. The solution was then irradiated with 365 nm light for 2 min, after which the sample was allowed to relax in the dark. Intermittent CD spectra were recorded to monitor the progress of the relaxation/induction. After full relaxation, 7 µL of the sample was injected into the HPLC to measure the enantiomeric ratio using the same method as described in Section 1.3.

Determination of Racemization Barriers

A solution of (*P*)-**BTX-21c7** (~50 µM in 1,2-dichloroethane) was stirred at 80 °C in the spectropolarimeter for ~4 h. CD spectra between 450 and 250 nm were measured periodically. A fast scanning speed of 500 nm/min and closing the shutter in between measurements was necessary to reduce the amount of light-induced racemization. Following the time-dependent decay curve of the ellipticity at 290 nm allowed determining the racemization barrier (Figures S19-20).

(*M*)-**BTX-21c7**$^{2+}$ was obtained by adding an excess of $Fe(ClO_4)_3 \bullet xH_2O$ (in a minimal amount of MeCN) to a solution of (*M*)-**BTX-21c7** (~50 µM in 1,2-dichloroethane). Full conversion to the dication was confirmed by UV-Vis spectroscopy. The obtained solution of (*M*)-**BTX-21c7**$^{2+}$ (~50 µM in 1,2-dichloroethane) was then stirred at 80 °C in the spectropolarimeter for 3 h, while constantly measuring the ellipticity at 295 nm. The time-dependent ellipticity revealed stable chiroptical properties at 80 °C, indicating no observable racemization at this temperature (Figure S23)

1.5 UV-Vis Measurements

UV-Vis spectra were recorded on an Agilent Cary 8454 spectrophotometer. All optical measurements were carried out using 1 cm pathlength quartz cuvettes. Measurements of the *syn*-folded state (or its relaxation) were carried out by irradiation of a stirred cuvette with a Thorlabs LED (M365) at a 90° angle which enabled rapid and quantitative photoswitching.

The relaxation speed of the thermal back isomerization was followed by UV-vis absorbance measurements of a 50 µM solution of **BTX-21c7** in CH_2Cl_2 at different concentrations of *(R)*-

MDBA which was achieved by addition of aliquots of the cation salt (50 mM in CH_2Cl_2 also containing 50 µM of the BTX-crown ether).

A UV-Vis calibration curve (Figure S3) of **BTX-21c7** was obtained by measuring spectra at varying known concentrations of **BTX-21c7**. This allowed determining the concentration of dilute solutions where only small quantities were available (e.g. for enantiopure **BTX-21c7**).

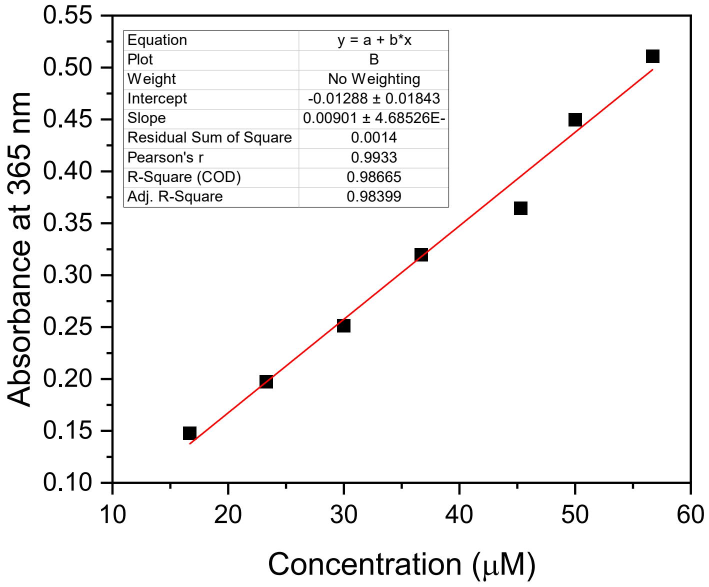

Figure S3. Absorbance of *rac*-**BTX-21c7** at λ = 365 nm at different concentrations in CH_2Cl_2. The linear fit was used as calibration curve.

1.6 ^{1}H NMR Irradiation/Titration Studies

NMR irradiation/titration experiments were carried out using a Varian Inova 500 spectrometer. All NMR titrations were carried out in CD_2Cl_2 at an initial **BTX-21c7** concentration of 1 mM at 25 °C. For titrations without any irradiation, an initial host volume of 500 µL was used, into which the guest was titrated as a 50 mM stock solution in CD_2Cl_2 (0.25 to 7 equivalents). Samples were kept in the dark in between the additions in order to prevent induction/racemization during the experiment.

For titration of the *syn*-folded state, the sample was also irradiated *in situ* with 365 nm light using a Thorlabs LED (M365) with a 1500 µm optical fiber (FT1500UMT) to guide the light directly into the NMR tube. Spectra were then recorded under continuous irradiation. In this case an initial host volume of 400 µL was used, into which the guest was titrated as a 40 mM stock solution in CD_2Cl_2 (0.25 to 18 equivalents).

Isotherm fitting was carried out with Origin 2018 using a global fit of the shift of at least two different proton environments. In all cases 1:1 host-guest stoichiometric binding models were used.[4]

1.7 Spectroelectrochemistry

The redox cycling stability of **BTX-21c7** in the presence of the **MDBA** guest was probed by UV/vis-spectroelectrochemistry in an OTTLE cell with an optical path length of ~0.2 mm containing a Pt grid as both working and counter electrode and a Ag wire as a pseudo-reference electrode. Measurements

were conducted in CH_2Cl_2 containing 200 mM $TBAPF_6$, 1.35 mM **BTX-21c7** and 5 equiv. of **MDBA**. After recording a CV of this solution, repeat alternating potential-controlled bulk electrolysis was carried out at potentials that were at least 150 mV anodic, or cathodic of the peak potentials. Each cycle took ~45 s. During electrolysis, UV/vis spectra were recorded every 5 s using a fiber-optics spectrometer setup from Avantes (AVASPEC-HSC1024X58TEC-EVO spectrometer and AVALIGHT-DH-S-BAL light source). For the blank measurement, the empty cell was used.

1.8 Computational Methods

For every studied structure (*syn*- and (*P*/*M*)-*anti*-folded isomers of **BTX-21c7**, with and without a **MDBA** guest and the oxidized dicationic state of the host), ensembles of conformers were generated using the CREST[5-7] (Conformer–Rotamer Ensemble Sampling Tool) software at the GFN2-xTB(GBSA)[8] level of theory using CH_2Cl_2 as the solvent. An energy threshold of 20 to 100 kcal/mol was used, leading to ensembles composed of 3793 to 18721 conformers. For the cationic host-guest complexes, the counter anion was not considered. For the host-guest complexes, the NCI (non-covalent interaction) mode of CREST was used. For the *syn* isomers, a constraint was applied on the S-S distance (distance = 4.3 Å, force constant = 0.001).

These ensembles of conformers were further refined using the CENSO software[9] (Commandline Energetic Sorting) following a three-step workflow involving a cheap pre-screening with an energy threshold of 4 kcal/mol at the B97-D3/def2-SV(P) level of theory, using the Orca 5.0.4software[10]. Then, a more accurate energy calculation was performed at the r^2SCAN-3c/SMD[dichloromethane]/GmRRHO(GFN2[alpb]-bhess) level of theory, using Orca for calculating the single point energy while the thermostatistical contributions (G_mRRHO) were calculated using the XTB software. Following this step, conformers within a 3.5 kcal/mol energy difference from the lowest lying one were conserved for the next step. Finally, the geometry of all remaining isomers (ensembles of 21 to 115 conformers) was optimized with the composite method r^2SCAN-3c,[11] using the Solvation Model Density SMD(dichloromethane) solvent model[12] with Orca. The thermochemical data was calculated at 25 °C. Single-point energy was calculated at this level of theory, while G_mRRHO was calculated at the GFN2[alpb]-bhess level of theory.

Calculations at the time-dependent DFT level of theory (TD-DFT) were then performed with the ORCA 5.0.4 software, using the previously optimized geometries of the lowest-energy conformer, using the conductor-like polarizable continuum CPCM(CH_2Cl_2) solvent model[13] at the ωB97X-D3[14-15]/def2-TZVPP[16] level of theory without using the Tamm-Dancoff Approximation (no TDA).

2. Chiral Induction Control Experiments

Various control experiments were carried out using analogous experimental procedures as described for the chiral induction titration studies in section 1.3 or the low-temperature CD studies in section 1.4.

Mixing *rac*-**BTX-21c7** with (*R*)-**MDBA** in CH_2Cl_2 at room temperature without irradiation did not lead to an enantioenrichment (Figure S4).

<Chromatogram>

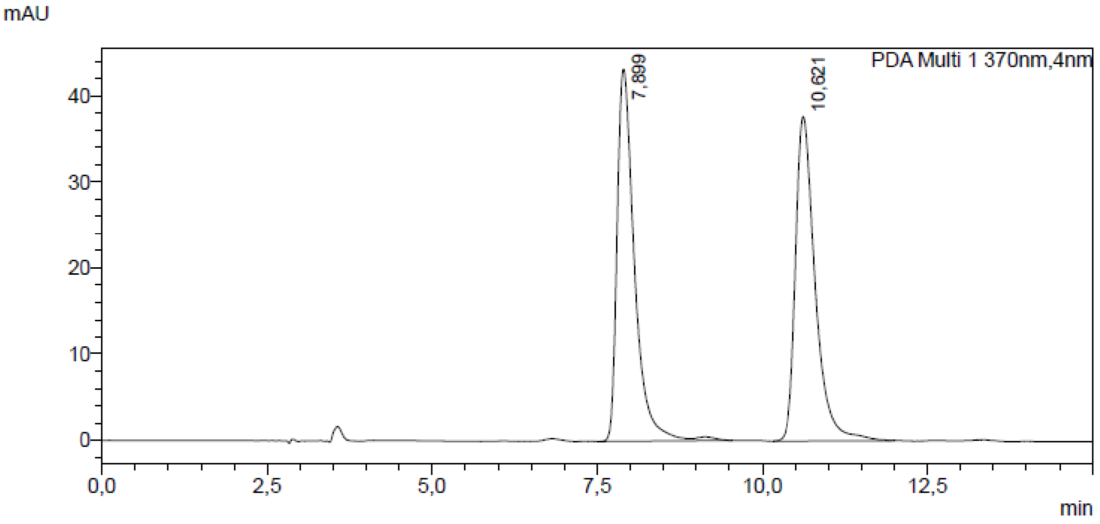

<Peak Table>

PDA Ch1 370nm

Peak#	Ret. Time	Area	Height	Conc.	Unit	Mark	Name
1	7,899	789886	43187	0,000			
2	10,621	781230	37678	0,000		S	
Total		1571116	80865				

Figure S4. HPLC chromatogram of a racemic mixture of **BTX-21c7** in presence of (*R*)-**MDBA** in CH_2Cl_2 without irradiation.

Mixing *rac*-**BTX-21c7** with (*R*)-**MDBA** in CH_2Cl_2, followed by irradiation with 365 nm and subsequent relaxation at room temperature led to significant enantioenrichment of (*M*)-**BTX-21c7** (Figure S5).

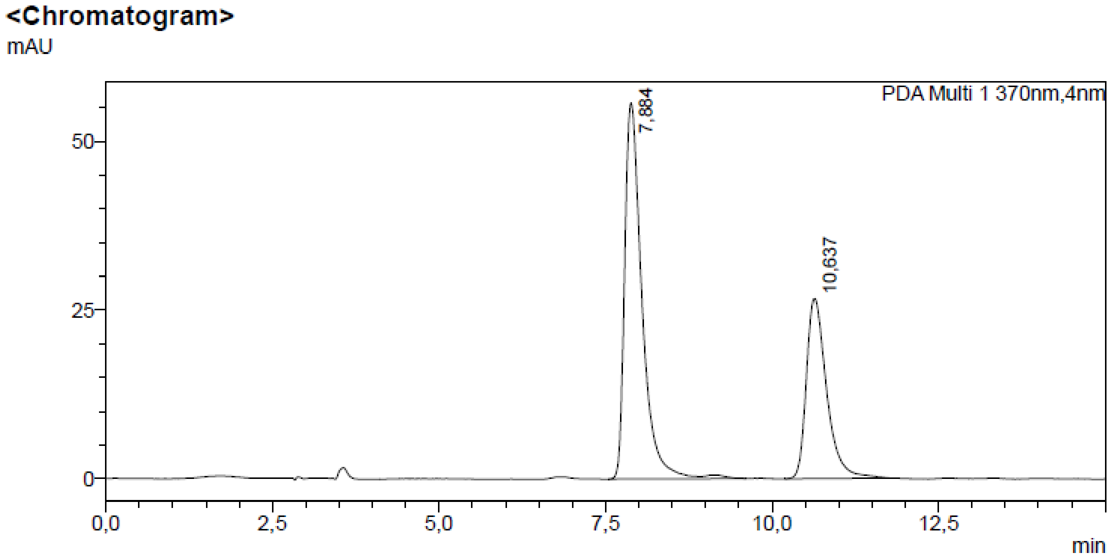

<Peak Table>

PDA Ch1 370nm

Peak#	Ret. Time	Area	Height	Conc.	Unit	Mark	Name
1	7,884	1018394	55823	0,000			
2	10,637	559259	26770	0,000			
Total		1577653	82593				

Figure S5. HPLC chromatogram after mixing *rac*-**BTX-21c7** and excess (*R*)-**MDBA** in CH_2Cl_2, followed by irradiation with 365 nm and subsequent relaxation at room temperature.

Even in the presence of 30 equiv. of the neutral, free base amino precursor (*R*)-N-benzyl-α-methylbenzylamine no induction was observed after an irradiation-relaxation cycle (Figure S6), confirming the necessity for formation of the complex between the host and the protonated ammonium guest.

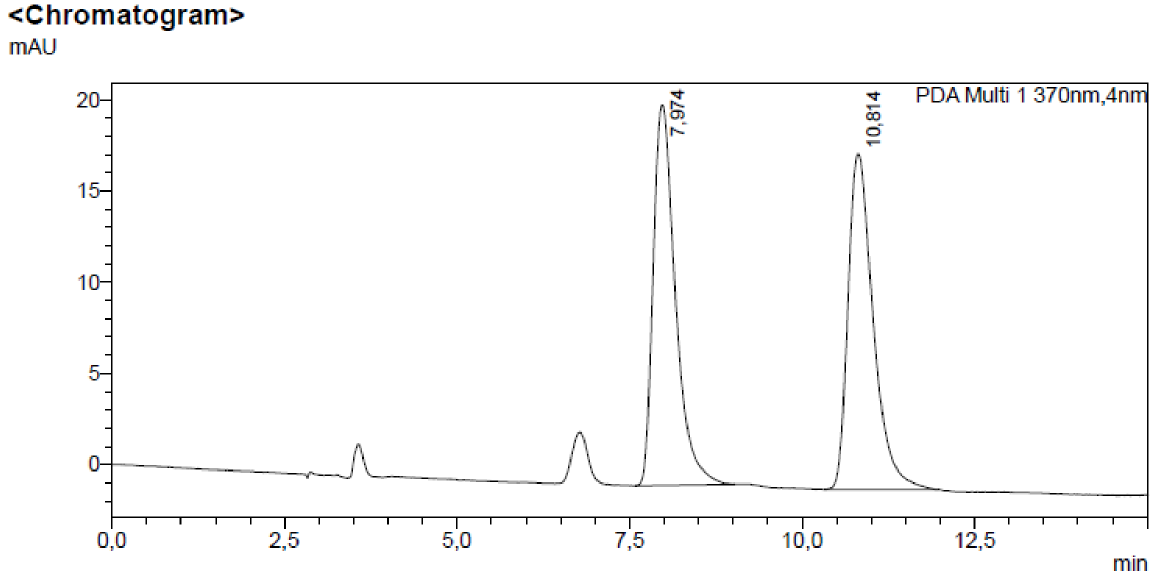

<Peak Table>

PDA Ch1 370nm

Peak#	Ret. Time	Area	Height	Conc.	Unit	Mark	Name
1	7,974	466802	20853	0,000			
2	10,814	467928	18410	0,000			
Total		934731	39263				

Figure S6. HPLC chromatogram after mixing *rac*-**BTX-21c7** and excess (*R*)-N-benzyl-α-methylbenzylamine in CH_2Cl_2, followed by irradiation with 365 nm and subsequent relaxation at room temperature.

Irradiation of enantiopure (*P*)-**BTX-21c7** with 365 nm followed by relaxation at room temperature resulted in the formation of a *rac*-**BTX** (Figure S7), confirming the proposed pathways via the prochiral *syn*-folded state **BTX-21c7**$_{\boldsymbol{syn}}$.

<Chromatogram>

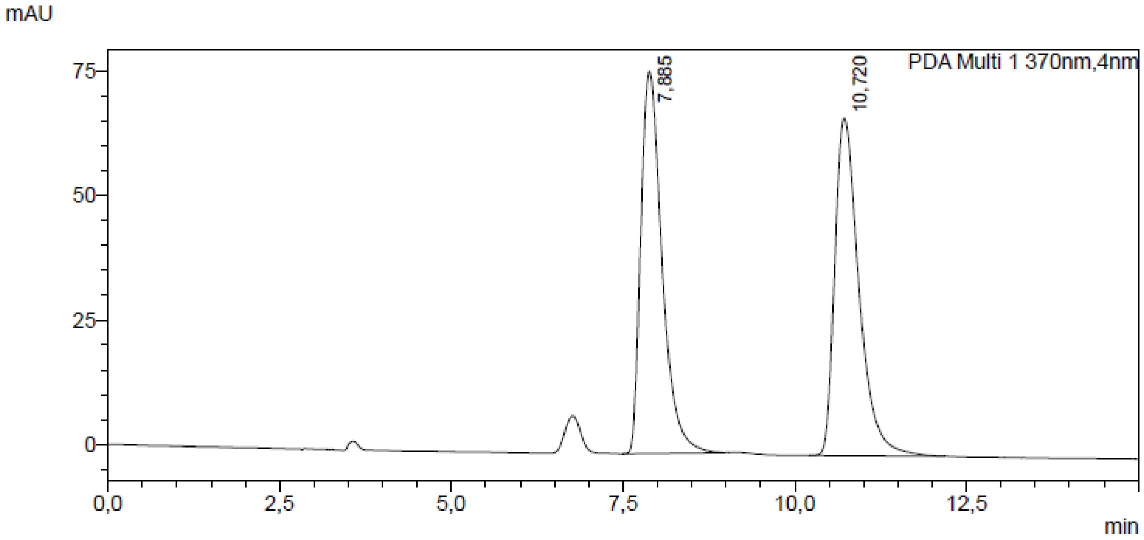

<Peak Table>

PDA Ch1 370nm

Peak#	Ret. Time	Area	Height	Conc.	Unit	Mark	Name
1	7,885	1648752	76707	0,000			
2	10,720	1669383	67781	0,000			
Total		3318135	144488				

Figure S7. HPLC chromatogram of a racemic mixture of **BTX-21c7** obtained by irradiating enantiopure (*P*)-**BTX-21c7** with 365 nm light, followed by relaxation at room temperature.

The outcome of the chiral induction is independent of the enantiopurity of the starting mixture of **BTX-21c7**, again confirming the pathway via the prochiral *syn*-folded state **BTX-21c$_{syn}$**. This is shown in Figure S8 and S9 where the chiral induction of enantiopure (*P*)-**BTX-21c7** with either (*R*)-**MDBA** or (*S*)-**MDBA** results in an opposite enantiomeric excess.

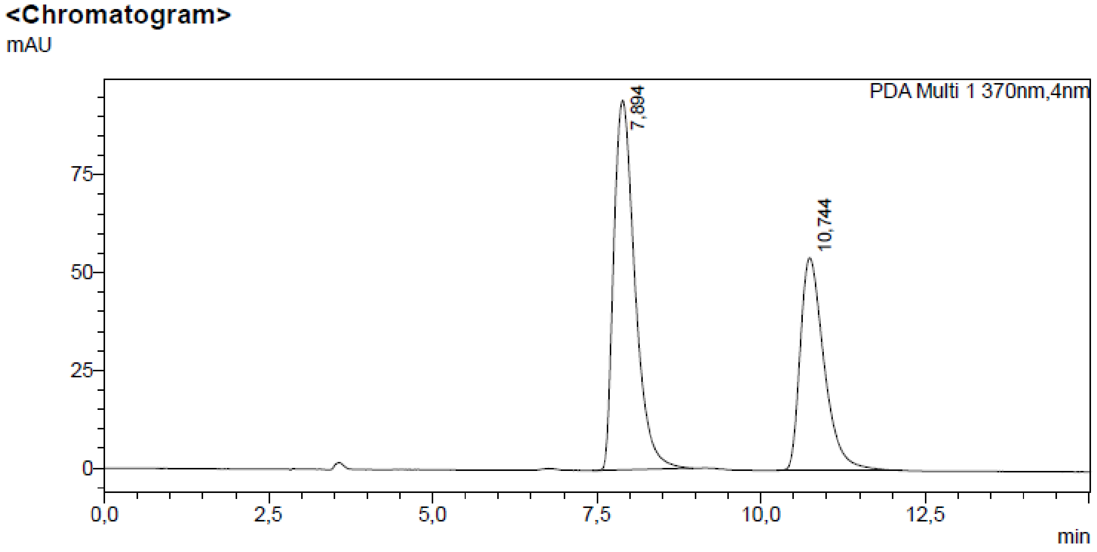

<Peak Table>

PDA Ch1 370nm

Peak#	Ret. Time	Area	Height	Conc.	Unit	Mark	Name
1	7,894	2103898	94490	0,000		M	
2	10,744	1371493	54273	0,000			
Total		3475391	148762				

Figure S8. HPLC chromatogram after chiral induction of enantiopure (*P*)-**BTX-21c7** with (*R*)-**MDBA**.

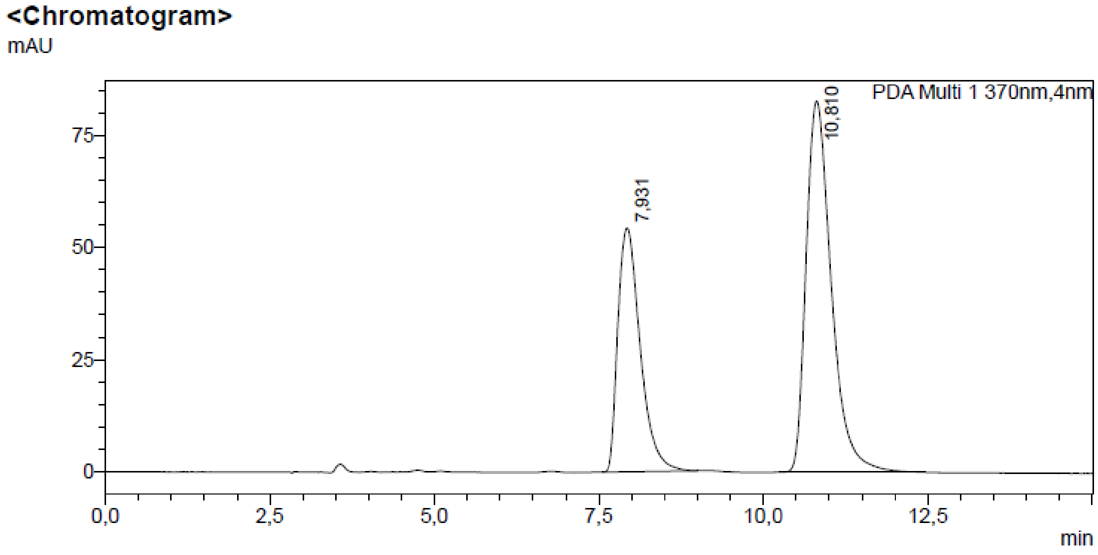

<Peak Table>

PDA Ch1 370nm

Peak#	Ret. Time	Area	Height	Conc.	Unit	Mark	Name
1	7,931	1274935	54358	0,000		V	
2	10,810	2199300	82670	0,000			
Total		3474235	137027				

Figure S9. HPLC chromatogram after chiral induction of enantiopure (*P*)-**BTX-21c7** with (*S*)-**MDBA**.

Adding (*R*)-**MDBA** (15 equivalents) to *rac*-**BTX-21c7** in CH_2Cl_2 while irradiating at 365 nm (more precisely, adding it to **BTX-21c7*syn***) resulted in the same enantioenrichment as when the guest is already present before irradiation (Figure S10).

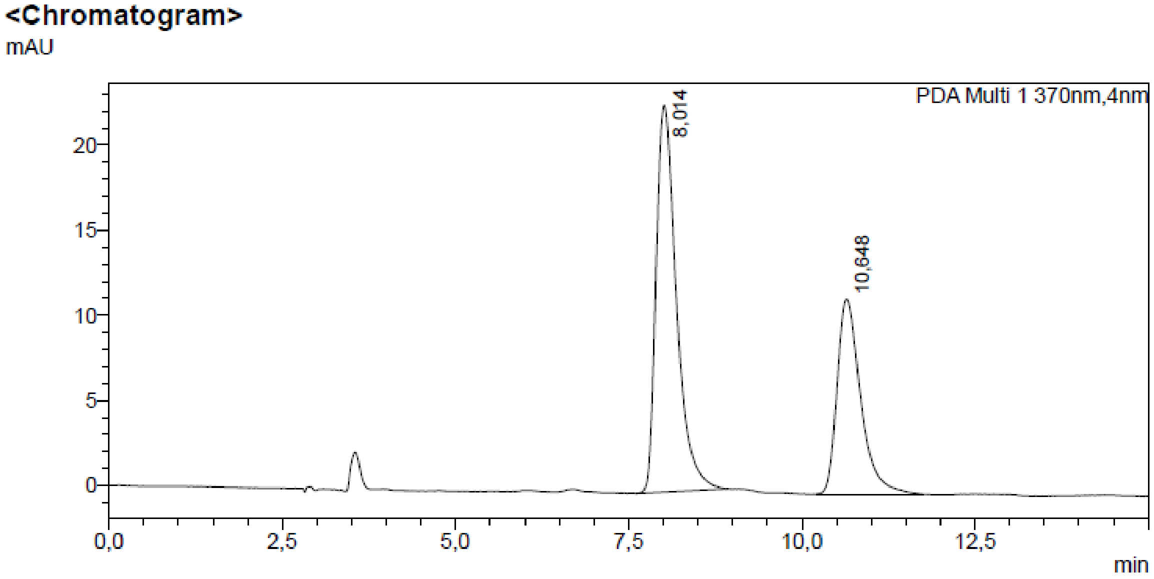

<Peak Table>

PDA Ch1 370nm

Peak#	Ret. Time	Area	Height	Conc.	Unit	Mark	Name
1	8,014	464042	22726	0,000			
2	10,648	272408	11456	0,000			
Total		736450	34181				

Figure S10. HPLC chromatogram of the chiral induction of *rac*-**BTX-21c7** after adding (*R*)-**MDBA** while irradiating to **BTX-21c7*syn***.

Reduction of **BTX-21c7^{2+}** (250 µM in CH_2Cl_2) with 3 equivalents of decamethylferrocene in presence of (*R*)-**MDBA** (15 equivalents) resulted in chiral induction and enantioenrichment of (*M*)-**BTX-21c7**, identical to the chiral induction with light (Figure S11).

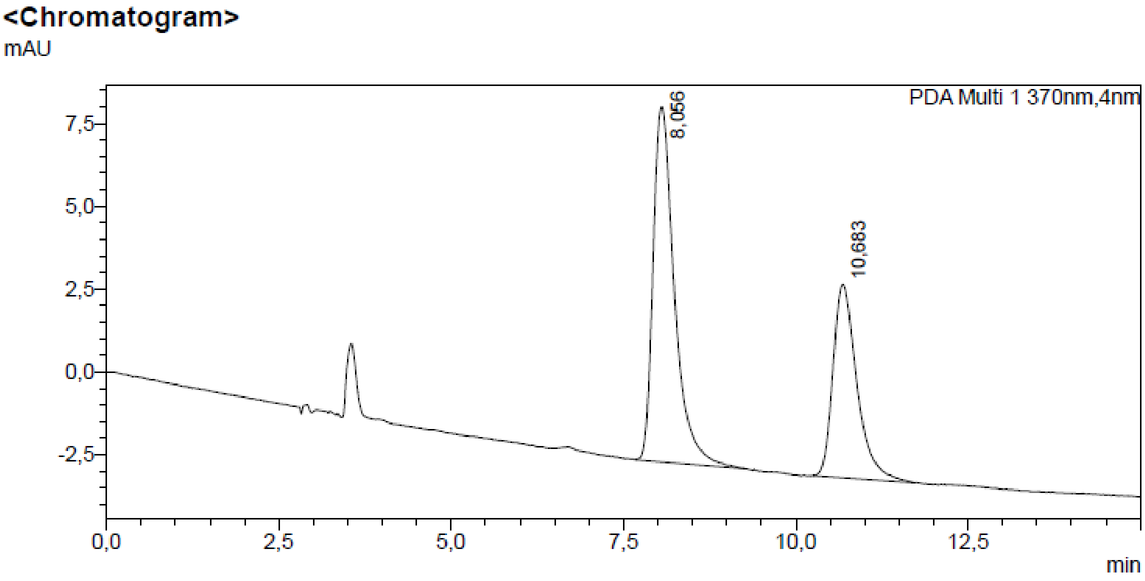

<Peak Table>

PDA Ch1 370nm

Peak#	Ret. Time	Area	Height	Conc.	Unit	Mark	Name
1	8,056	230702	10728	0,000			
2	10,683	140729	5836	0,000			
Total		371431	16564				

Figure S11. HPLC chromatogram of the chiral induction of **BTX-21c7^{2+}** with (*R*)-**MDBA** after reduction with decamethylferrocene in CH_2Cl_2.

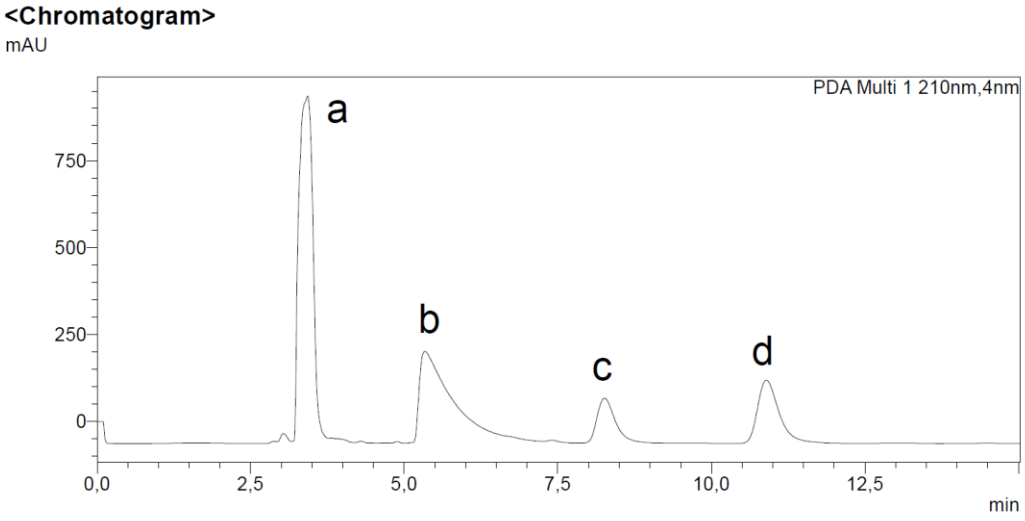

Figure S12. HPLC chromatogram after typical light-driven chiral induction of enantiopure (*P*)-**BTX-21c7** with (*S*)-**MDBA** using the absorbance read-out at 210 nm. Under these conditions the solvent injection peak (a) is more visible. Additionally, the MDBA guest absorbs in this region (peak b), highlighting that the guest can be chromatographically separated and that the host peaks (c and d) are those of the pure host enantiomers and not of the diastereomeric hostguest complexes.

3. Mechanistic Studies

Evidence for host guest interactions between **BTX-21c7** and **MDBA** was obtained by NMR experiments (vide infra) as well as HR-MS. Specifically, we observed a weak, but noticeable signal with the correct isotope pattern at m/z = 882.3498 which is in excellent agreement with the calculated m/z of 882.3493 for the host guest complex ($C_{53}H_{56}NO_7S_2$, $[M{-}PF_6^-]^+$). By analogy with related host-guest systems based on crown ethers and (M)DBA guests,[1, 17] we believe that binding of **MDBA** to **BTX-21c7** occurs via threading of the guest through the macrocycle. In this context we would also like to note that for the simpler, symmetric dibenzylammonium (DBA) guest, we recently showed that the same switch scaffold with a smaller crown ether (**BTX-18c6**) does not display any binding to the dibenzylammonium (DBA) cation,[3] which is strongly indicative that non-pseudo-interlocked binding modes (e.g. “perched” binding) are not energetically favoured in these systems, *i.e.* pseudo-rotaxanation is the only relevant binding mode between **BTX-21c7** and **MDBA**, as further confirmed by theoretical studies (see Figure 2b and Section S6).

^{1}H NMR titrations were then carried out to determine various relevant binding constants. As shown in Figures S13-14, titration of the enantiopure (*M*)-**BTX-21c7** with either (*R*)-**MDBA** or (*S*)-**MDBA** enabled determination of the binding constants of these diastereomeric complexes. Specifically, in both cases small, but significant shifts of multiple proton environments were observed (Figure S15). Fitting of two distinct aromatic proton environments to a 1:1 host-guest stoichiometric binding model via global fitting then revealed a higher stability of the (*R*)-**MDBA●**(*M*)-**BTX-21c7*anti*** complex ($K_{M\bullet R} = 26900 \pm 6900\ \mathrm{M}^{-1}$) than for the (*S*)-**MDBA●**(*M*)-**BTX-21c7*anti*** complex ($K_{M\bullet S} = 18700 \pm 2300\ \mathrm{M}^{-1}$). This corresponds to a binding free energy difference of ΔG = –24.86 and –23.97 kJ/mol, respectively.

Note also that due to their enantiomeric relationship, the binding constant for (*S*)-**MDBA** to (*P*)-**BTX-21c7*anti*** $K_{P\bullet S}$ is identical to $K_{M\bullet R}$, while $K_{M\bullet S} = K_{P\bullet R}$

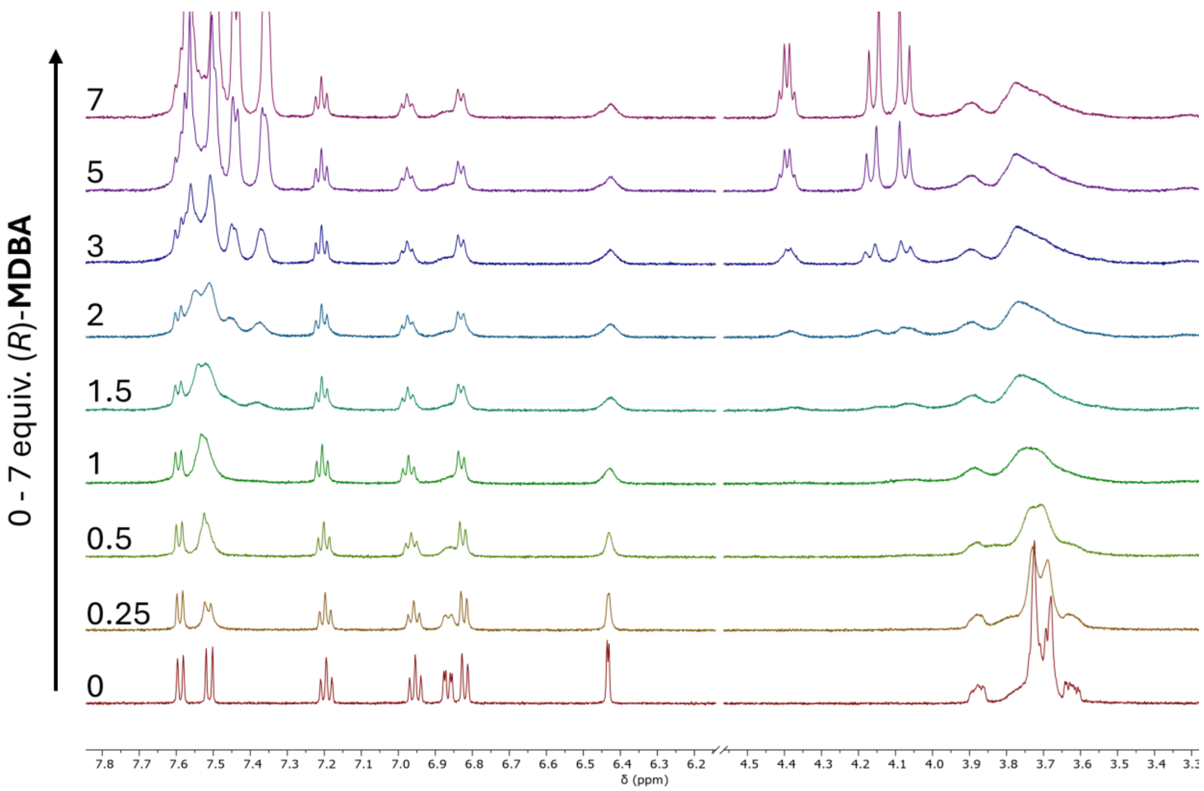

Figure S13. Stacked, partial ^{1}H NMR spectra of 1 mM (*M*)-**BTX-21c7** upon titration with increasing equivalents of (*R*)-**MDBA** (500 MHz in CD_2Cl_2, 25 °C).

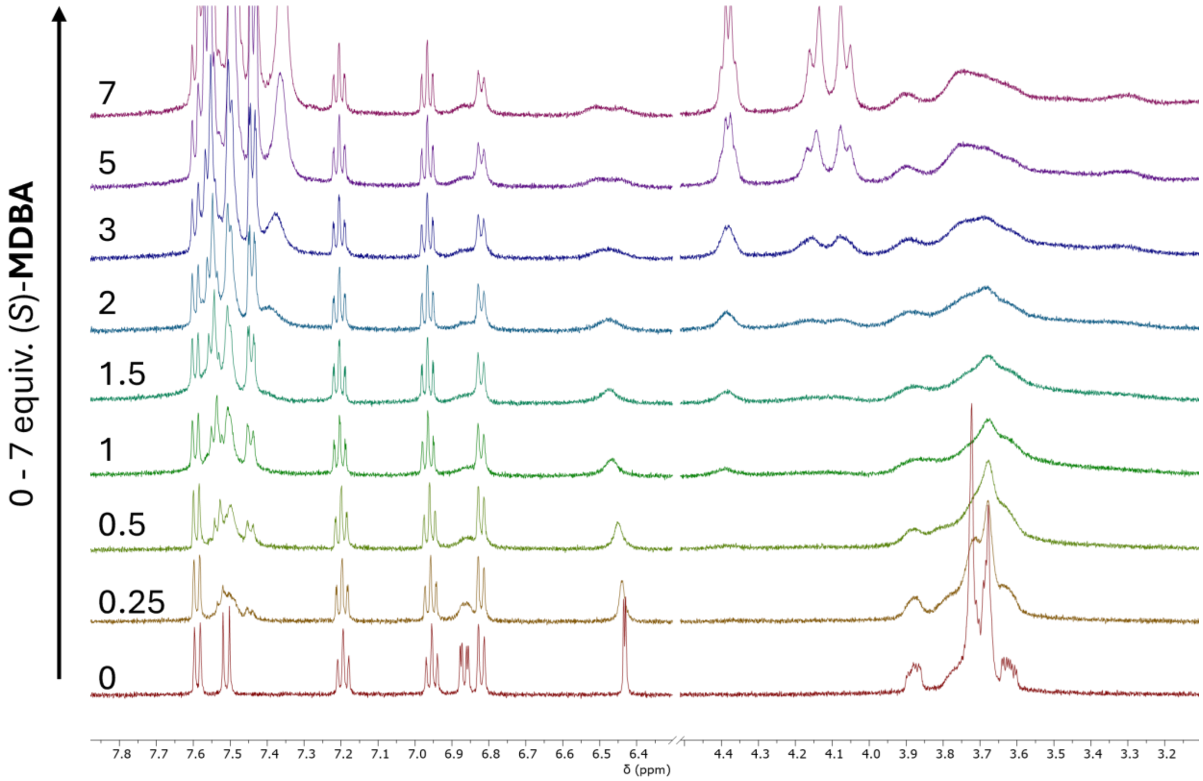

Figure S14. Stacked, partial ^{1}H NMR spectra of 1 mM (*M*)-**BTX-21c7** upon titration with increasing equivalents of (*S*)-**MDBA** (500 MHz in CD_2Cl_2, 25 °C).

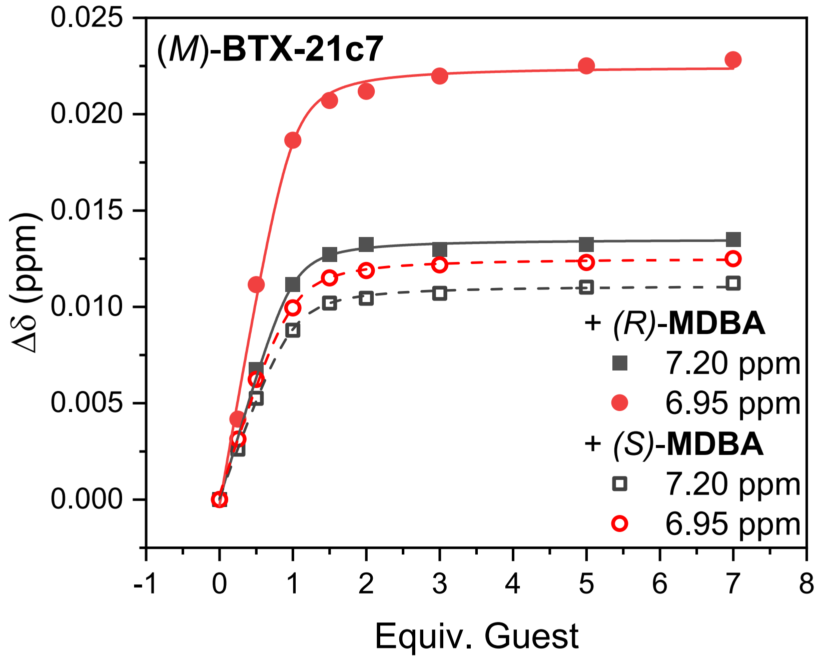

Figure S15. Change in chemical shift of two selected aromatic proton resonances of 1 mM (*M*)-**BTX-21c7** upon titration with increasing equivalents of (*R*)-**MDBA** (solid symbols) or (*S*)-**MDBA** (empty symbols) (500 MHz in CD_2Cl_2, 25 °C). The lines represent fits to a 1:1 host-guest stoichiometric binding model.

^{1}H NMR titrations were also conducted to determine the binding constant of the ammonium guest to the *syn*-folded receptor state. This was carried out by *in situ* NMR irradiation as described in Section 1.6. As shown in Figure S16, this enabled quantitative switching to the *syn*-folded state. In this case, only one titration with (*R*)-**MDBA** was carried out, as no diastereomeric complexes are formed due to the achiral nature of the *syn*-folded state. Due to weaker binding, up to 18 equiv. of the guest were added. Again, notable shifts of multiple proton resonances were observed (Figure S17). Fitting of the corresponding isotherms revealed much weaker binding to the syn-folded state of K_{syn} = 850 ± 40 M^{-1}, which corresponds to ΔG = –16.44 kJ/mol.

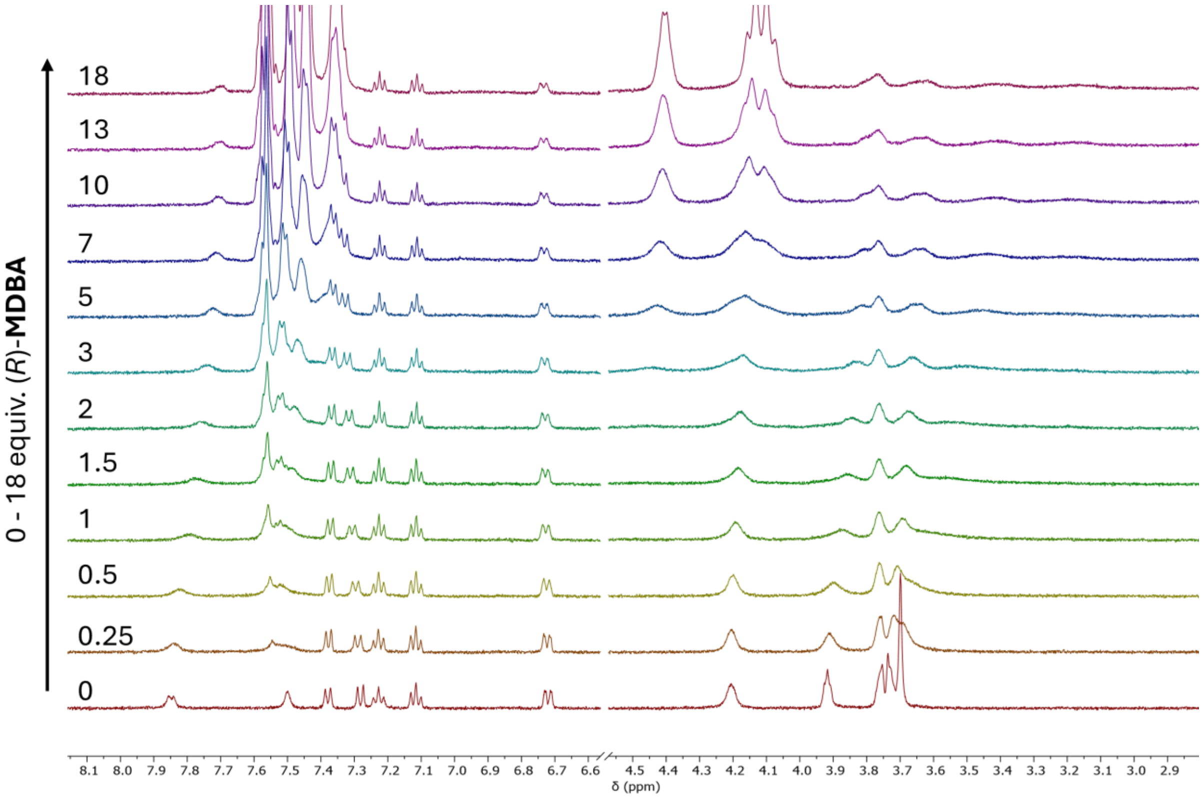

Figure S16. Stacked, partial ^{1}H NMR spectra of 1 mM **BTX-21c7**$_{syn}$, generated *via in situ* irradiation, upon titration with increasing equivalents of (*R*)-**MDBA** (500 MHz in CD_2Cl_2, 25 °C).

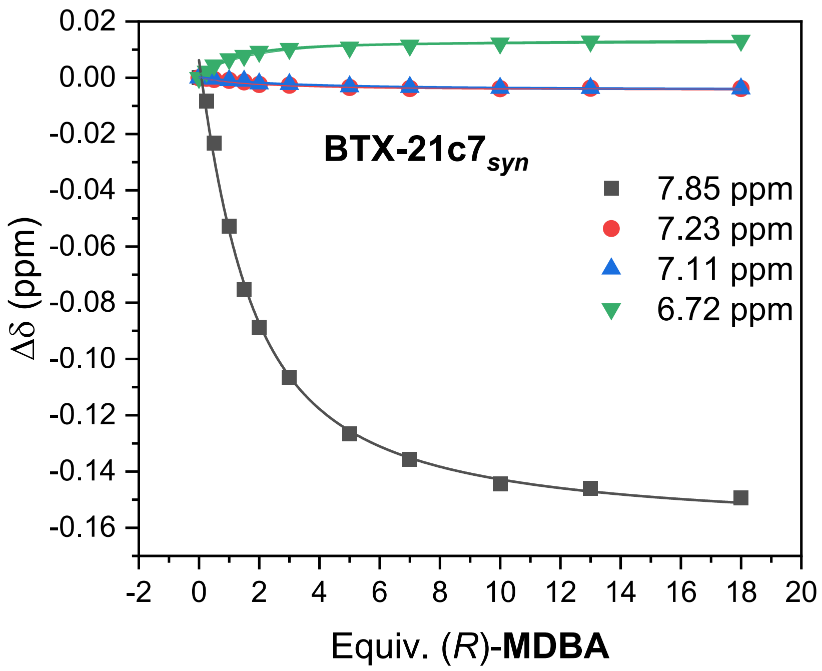

Figure S17. Change in chemical shift of four selected aromatic proton resonances of 1 mM **BTX-21c7*syn*** upon titration with increasing equivalents of (*R*)-**MDBA** (500 MHz in CD_2Cl_2, 25 °C). The solid lines represent fits to a 1:1 host-guest stoichiometric binding model.

As shown in Figures S18 and S19, in the presence of increasing concentrations of *(R)*-**MBDA** the thermal relaxation of the *syn*-folded state of **BTX-21c7** back to the *anti*-folded state is significantly sped up. Specifically, in the absence of the guest, the thermal half-life is ~65 s, which drops to ~30 s in the presence of excess guest. This observation is in good agreement with stronger guest binding in the anti-folded state and the Bell–Evans–Polanyi principle.[3] Furthermore, this observation highlights the simplicity and utility of the light-triggered induction process; as both light switching as well as relaxation are very fast, the overall induction process can be carried out in a few minutes in total.

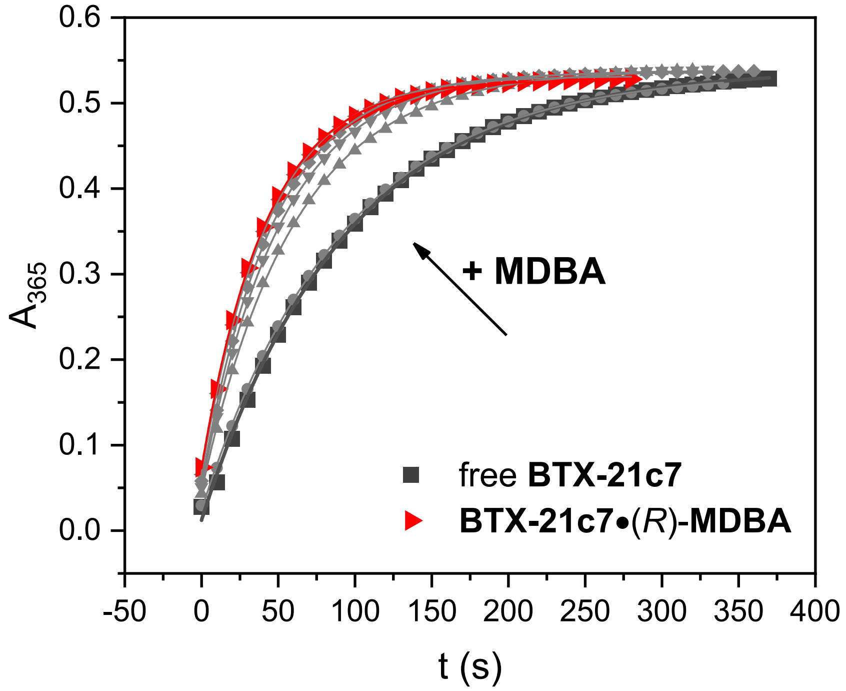

Figure S18. Change in absorbance at λ = 365 nm of **BTX-21c7** (50 µM in CH_2Cl_2, 25 °C), during thermal back relaxation from the *syn*- to the *anti*-folded state in the presence of increasing concentrations of (*R*)-**MDBA**. The solid lines correspond to a linear fit to a simple exponential decay, from which the $t_{1/2}$ was obtained (see also Figure S19).

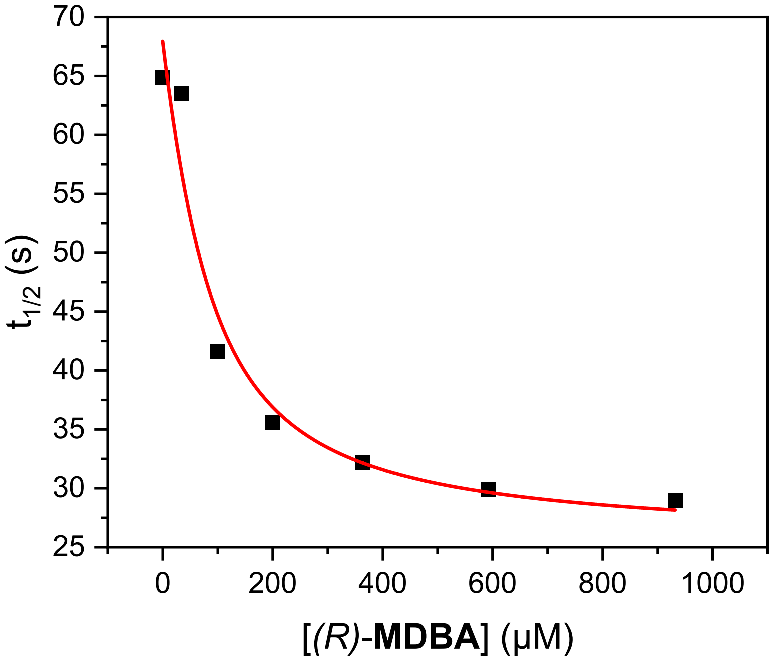

Figure S19. Change in thermal half-life $t_{1/2}$ for the *syn* –> *anti* relaxation of **BTX-21c7** (50 µM in CH_2Cl_2) as a function of *(R)*-**MBDA** concentration as determined by UV-vis measurements (at 25 °C).

Stirring a mixture of *rac*-**BTX-21c7** and (*R*)-**MDBA** in 1,2-dichloroethane at 80 °C for 3.5 h resulted in enantioenrichment of (*M*)-**BTX-21c7** with an *er* of 57:43 (Figure S20), indicating a binding energy difference of both diastereomeric complexes (*R*)-**MDBA●**(*M*)-**BTX-21c7** and (*R*)-**MDBA●**(*P*)-**BTX-21c7** of $\Delta\Delta G = \Delta G_{R\bullet M} - \Delta G_{R\bullet P} = -0.83\ \mathrm{kJ/mol}$) at 80 °C according to $\Delta G = -RTln(K)$ and $K = \frac{[R\bullet M]}{[R\bullet P]}$.

<Chromatogram>

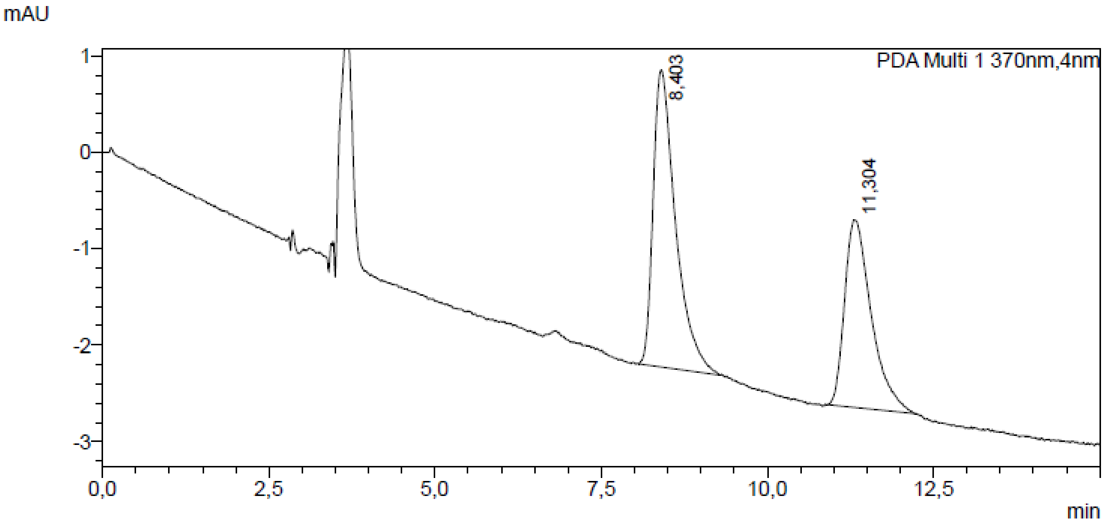

<Peak Table>

PDA Ch1 370nm

Peak#	Ret. Time	Area	Height	Conc.	Unit	Mark	Name
1	8,403	72115	3078	0,000			
2	11,304	54418	1942	0,000			
Total		126533	5020				

Figure S20. HPLC chromatogram after thermal chiral induction of *rac*-**BTX-21c7** and (*R*)-**MDBA** in 1,2-dichloroethane at 80 °C for 3.5 h. Completion of the thermal induction was confirmed by subsequent continuous heating and analysis by HPLC.

4. Determination of Racemization Barriers of BTX-21c7 and BTX-21c7^{2+}

The racemization barriers/properties of **BTX-21c7*anti*** and **BTX-21c7^{2+}** were determined by CD measurements at 80 °C in 1,2-dichloroethane.

Upon heating of (*P*)-**BTX-21c7*anti*** well defined decay of the CD signal was observed (Figure S21). Analysis of the exponential decay (Figure S22) allowed determining a half-life time of $t_{1/2}$ = 42.05 min at 80 °C. From this the racemization barrier was obtained using the Eyring equation and assuming a transmission coefficient of 1; ΔG_{rac} = 26.55 kcal/mol (111.1 kJ/mol) for **BTX-21c7*anti***. At 20 °C this corresponds to a half-life of $t_{1/2}$ = 82 d.

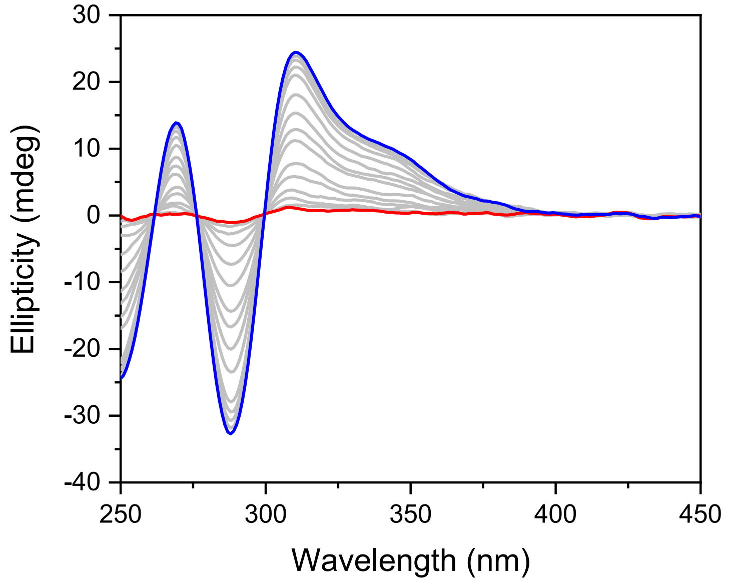

Figure S21. Blue: CD spectra of enantiopure (*P*)-**BTX-21c7** (~50 µM in 1,2-dichloroethane) at 80 °C. Grey: Periodically measured CD spectra of scalemic mixtures of **BTX-21c7** at 80 °C. Red: CD spectra of a near-racemic mixture of **BTX-21c7** at 80 °C after stirring for 4 h at 80 °C.

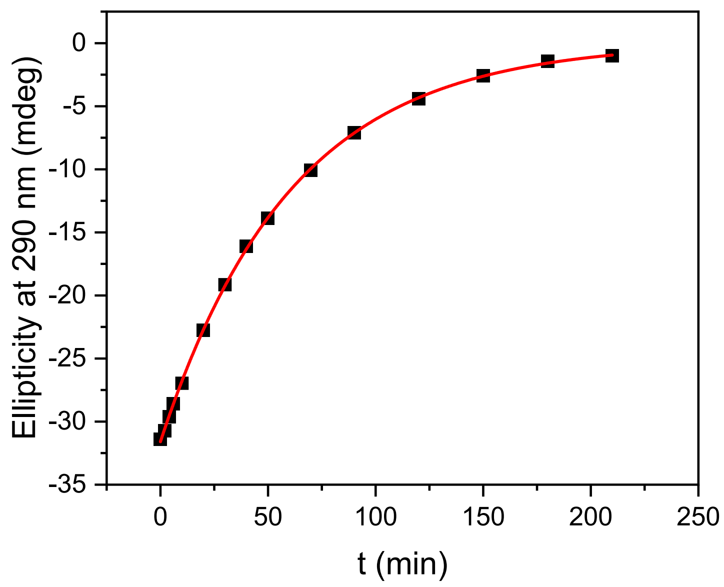

Figure S22. Ellipticity at 290 nm for periodically measured CD spectra of scalemic mixtures of **BTX-21c7** at 80 °C (~50 µM in 1,2-dichloroethane). The red solid line corresponds to a fit of a simple exponential decay.

We also attempted to determine the racemization barrier of the dicationic state in the same manner. To this end, (*M*)-**BTX-21c7^{2+}** was generated *in situ* by oxidation of (*M*)-**BTX-21c7** with $Fe(ClO_4)_3$●xH_2O in 1,2-dichloroethane. However, in this case no significant decay of the CD signal was observed even after heating at 80 °C for 3 h (Figure S23). As such, no racemization barrier could be determined, however this confirms that **BTX-21c7^{2+}** has a very high configurational stability. As a result, the chiral information is stored even more permanently in this state and can also not be erased upon irradiation with light, but only by reduction.

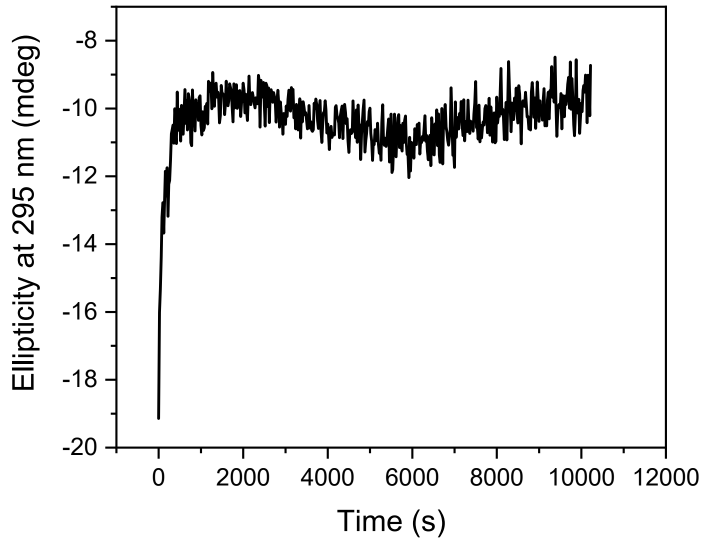

Figure S23. Time-dependent ellipticity at 295 nm of (*M*)-**BTX-21c7^{2+}** (~50 µM in 1,2-dichloroethane) at 80 °C, displaying a stable CD signal at this temperature for ~3 h. The initial decay of CD signal from –19 to –10 mdeg is due to thermal CD changes and not due to racemization. Specifically, the full CD spectrum at room temperature at the beginning of the experiment and at the end of the time-course measurement (after cooling back to rt) were identical (with an ellipticity of –19 mdeg at 295 nm), indicating no observable racemization.

5. Cycling Experiments

The fatigue resistance of light-driven as well as redox switching of the **BTX-21c7** was probed by UV/vis (Figures S24-25), HPLC (Figure S26) and spectroelectrochemistry (Figures S27-29), which confirm a high degree of cyclability under ambient conditions in both cases.

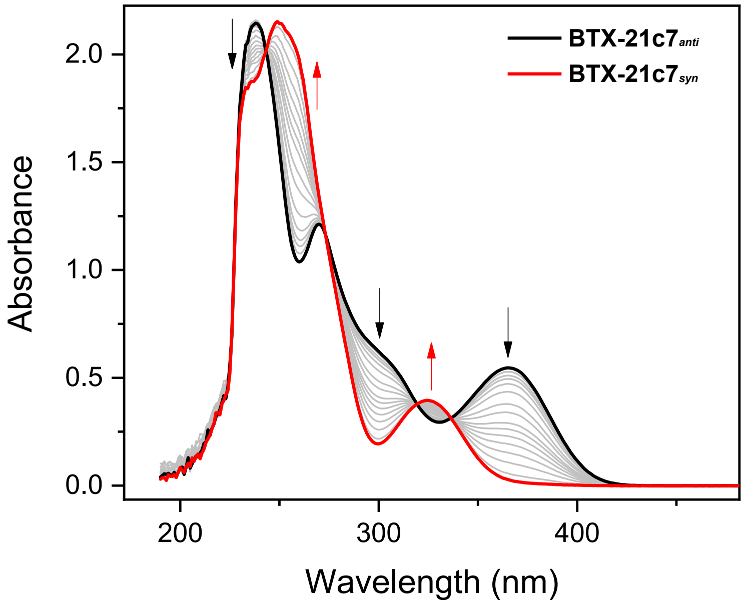

Figure S24. Changes in UV/vis spectrum of 50 µM **BTX-21c7** in DCM upon light-driven switching (365 nm light) from the *anti*-folded state (black) to the *syn*-folded state (red). Both the forward switching as well as the purely thermal relaxation (not shown here) occur via the same absorbance changes with clear isosbestic points.

As shown in Figure S25, the photoswitching/thermal relaxation cycle between the *anti*- and *syn*-folded states could be repeated for 13x without noticeable changes/degradation. However, we would like to note that prolonged irradiation with high energy light (especially deep UV light) sometimes induces a small degree of oxidation to the dicationic state, especially in the presence of the MBDA cation. This is presumably because the cation contains residual acid which can act as an oxidizing agent, and because the oxidation potential of the *syn*-folded state is lower than that of the *anti*-folded state. Nevertheless, even if the dicationic state is formed, this can be easily reduced back, i.e. the system does not overall degrade.

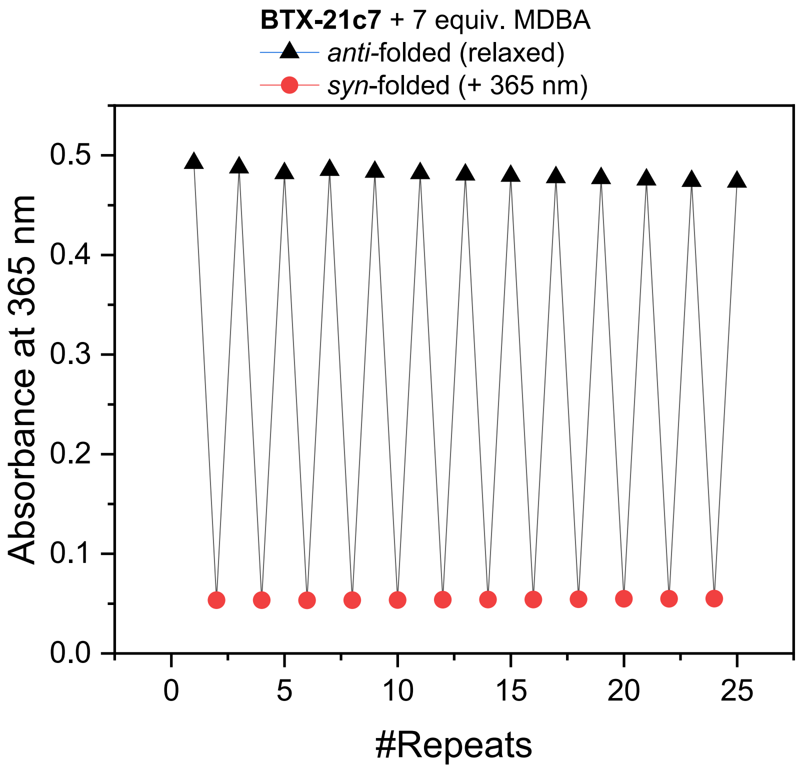

Figure S25. Repeat light-driven interconversion between the *anti*- (black triangles) and *syn*-folded (red circles) states of **BTX-21c7** (50 µM in DCM) in the presence of 7 equiv. of MDBA as followed by UV/vis spectroscopy. Quantitative switching to the *syn*-folded state was achieved by irradiation with 365 nm after which the absorbance was measured under continuous irradiation. Back-switching to the *anti*-folded state is a purely thermal process that was completed within a few minutes after turning of the LED.

To confirm that this fatigue-resistance extends to the chiral induction process, we also probed the *ee* of the host by HPLC upon repeat irradiation/relaxation (Figure S26). For experimental reasons this was carried out on 7 identical samples, which were each subjected to an increasing number of full irradiation/relaxation cycles. Each irradiation was carried out for 2 min (i.e. sample 7 was irradiated for 14 min total), while relaxation was at least 3 min in each case. As shown in Figure S26, no notable change in ee as a function of irradiation time/number of cycles was observed. This further confirms that induction occurs in the electronic ground state and is not additive.

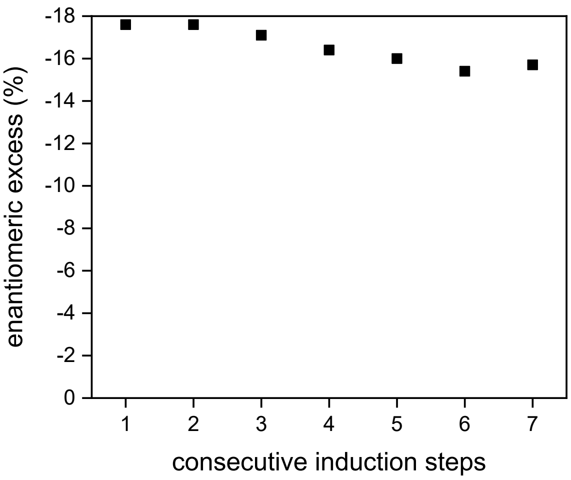

Figure S26. Enantiomeric excess of light-driven chiral induction of **BTX-21c7** (250 µM in DCM) in the presence of a small excess of (*S*)-**MDBA** upon 7 repeat irradiation/relaxation cycles as determined by HPLC.

Shown in Figure S27 is the partial CV of 1.35 mM **BTX-21c7** and 5 equiv. of **MDBA** in CH_2Cl_2 containing 200 mM $TBAPF_6$ under thin layer conditions in an OTTLE spectroelectrochemical cell. In good agreement with expectations, a single two-electron oxidation was observed at high potentials (+1.075 V vs Ag wire pseudo-reference), corresponding to the conversion from the *anti*-folded state of **BTX-21c7** to the dication **BTX-21c7^{2+}**. Two-electron reduction occurs at a much lower potential (+0.2 V, i.e. with large hysteresis of 0.875 V) and initially quantitatively generates the neutral *syn*-folded state of **BTX-21c7** which then thermally relaxes to the *anti*-folded state. For more detailed discussions on these redox/switching properties the interested reader is referred to our previous work.[3]

Figure S28 shows the absorbance spectrum of the pure **BTX-21c7^{2+}** dication (electrochemically generated) and the resultant spectral changes upon its electrochemical reduction.

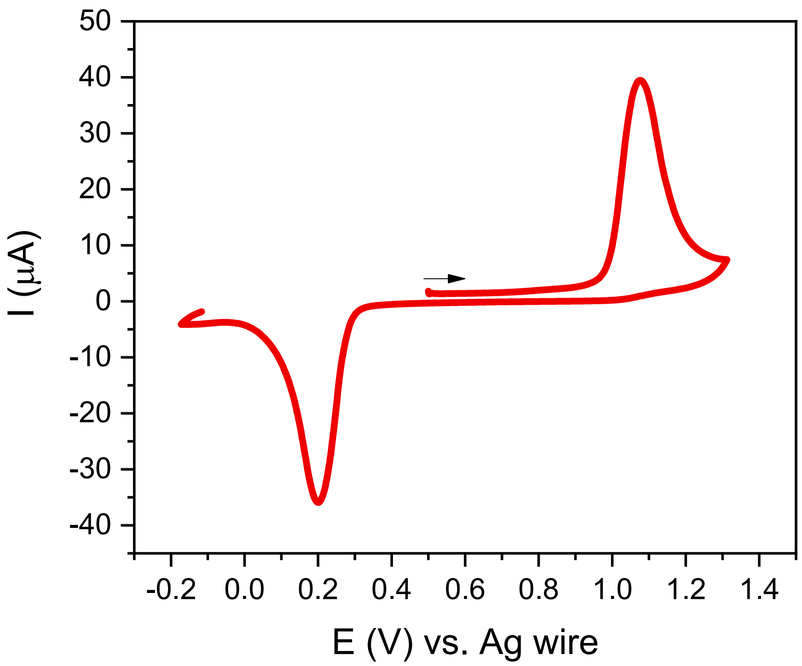

Figure S27. Partial CV of 1.35 mM **BTX-21c7** and 5 equiv. of **MDBA** in CH_2Cl_2, 200 mM $TBAPF_6$ under thin layer conditions at a scan rate of 5 mV/s. The black arrow indicates the starting point and initial direction of the scan.

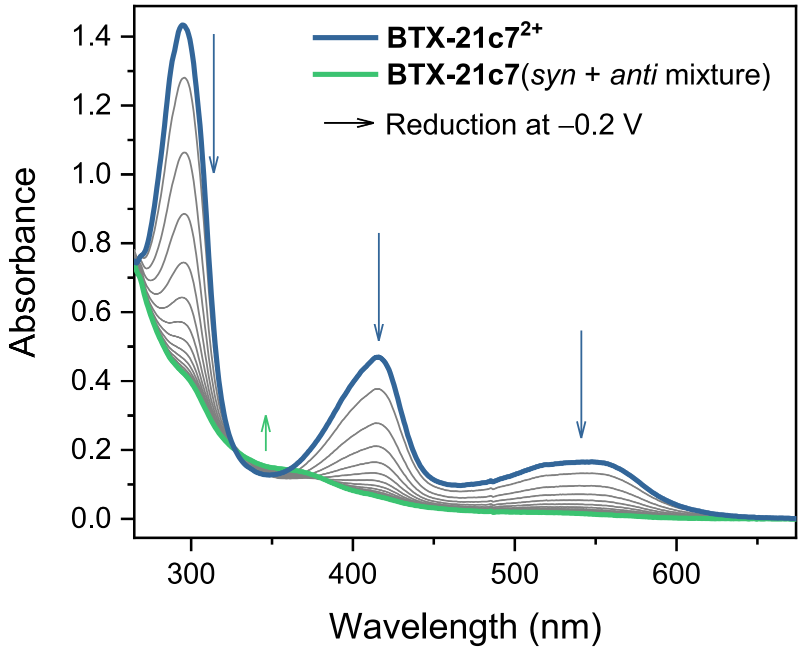

Figure S28. Changes in absorbance of 1.35 mM **BTX-21c7** and 5 equiv. of **MDBA** in CH_2Cl_2, 200 mM $TBAPF_6$ upon potential-controlled reduction of **BTX-21c7**$^{2+}$ (blue line, prior generated via exhaustive potential-controlled oxidation). This reduction cleanly forms first the *syn*-folded state, which then thermally relaxes to the *anti*-folded state. As a result, during the spectroelectrochemical experiment, a mixture of an unknown ratio of both neutral states is present under these conditions. Scans were recorded every 5 seconds.

As shown in Figure S29, repeat potential-controlled bulk electrolysis at +1.3 V and −0.2 V induces highly reversible changes in the UV/vis spectrum over many cycles without any apparent degradation. This confirms that redox-switching, under ambient conditions, is highly fatigue-resistant, even in the presence of the **MDBA** guest.

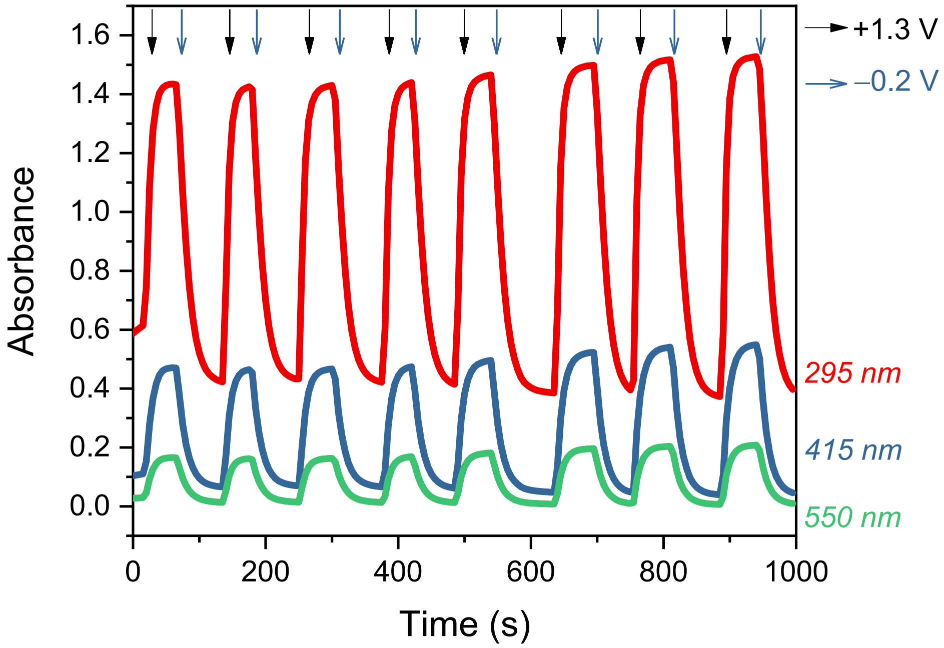

Figure S29. Changes in absorbance of 1.35 mM **BTX-21c7** and 5 equiv. of **MDBA** in CH_2Cl_2, 200 mM $TBAPF_6$ upon repeat bulk electrochemical redox cycling in a thin layer spectroelectrochemical cell at different selected wavelengths. The arrows indicate the points at which the potentials were switched.

6. Calculated Host-Guest Structures and Energies

The calculation procedure described in SI Section 1.7 yielded ensembles of conformers for the isomers of **BTX-21c7** with and without (*R*/*S*)-**MDBA** guests considered in this study. The geometry of the lowest-energy conformer for all the calculated ensembles is displayed in Figures S30-38 below.

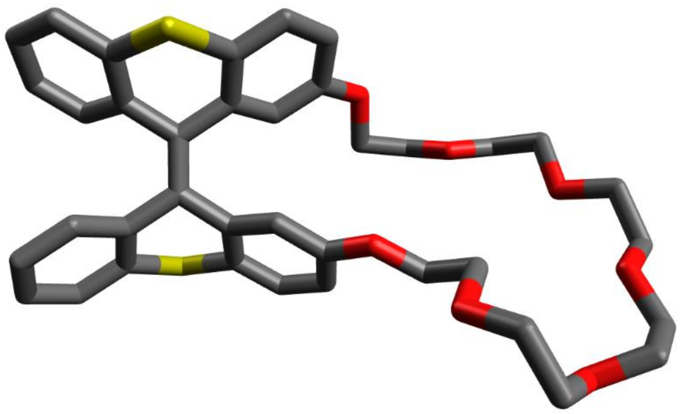

Figure S30. DFT-optimized structure for the lowest-energy conformer of (*P*)-**BTX-21c7*anti***. All hydrogen atoms are omitted for clarity. The optimization and ranking workflow are detailed in Section S1.7. Final geometry optimization: r^{2}scan-3c/SMD(CH_2Cl_2).

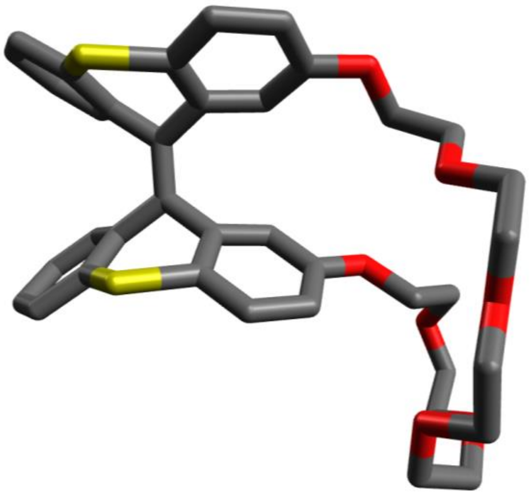

Figure S31. DFT-optimized structure for the lowest-energy conformer of **BTX-21c7*syn***. All hydrogen atoms are omitted for clarity. The optimization and ranking workflow are detailed in Section S1.7. Final geometry optimization: r^{2}scan-3c/SMD(CH_2Cl_2).

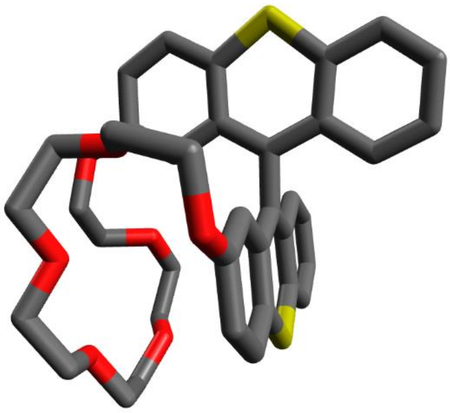

Figure S32. DFT-optimized structure for the lowest-energy conformer of (*M*)-**BTX-21c7^{2+}**. All hydrogen atoms are omitted for clarity. The optimization and ranking workflow are detailed in Section S1.7. Final geometry optimization: r^{2}scan-3c/SMD(CH_2Cl_2).

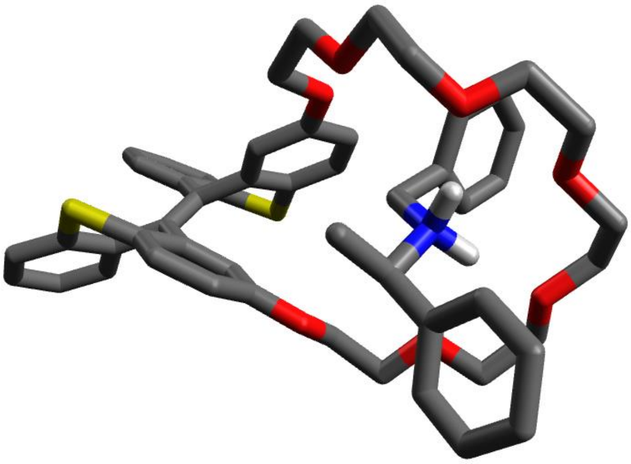

Figure S33. DFT-optimized structure for the lowest-energy conformer of (*R*)-**MDBA●**(*M*)-**BTX-21c7*anti***. Most hydrogen atoms are omitted for clarity. The optimization and ranking workflow are detailed in Section S1.7. Final geometry optimization: r^{2}scan-3c/SMD(CH_2Cl_2).

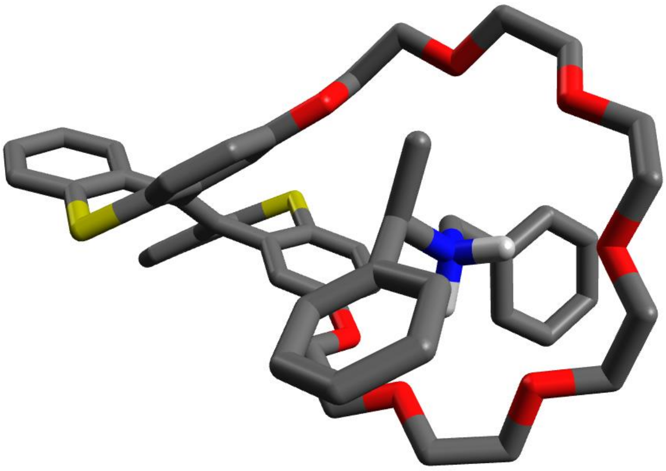

Figure S34. DFT-optimized structure for the lowest-energy conformer of (*R*)-**MDBA●**(*P*)-**BTX-21c7*anti***. Most hydrogen atoms are omitted for clarity. The optimization and ranking workflow are detailed in Section S1.7. Final geometry optimization: r^{2}scan-3c/SMD(CH_2Cl_2).

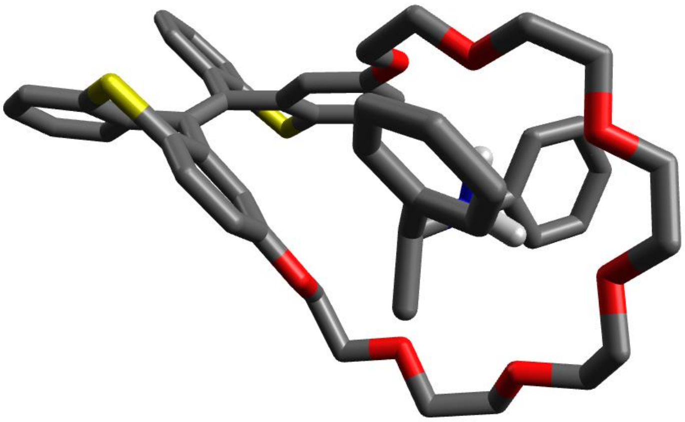

Figure S35. DFT-optimized structure for the lowest-energy conformer of (*S*)-**MDBA●**(*M*)-**BTX-21c7*anti***. Most hydrogen atoms are omitted for clarity. The optimization and ranking workflow are detailed in Section S1.7. Final geometry optimization: r^2scan-3c/SMD(CH_2Cl_2).

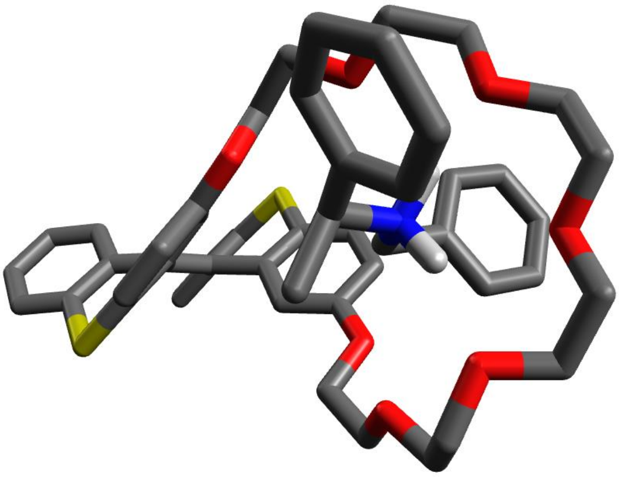

Figure S36. DFT-optimized structure for the lowest-energy conformer of (*S*)-**MDBA●**(*P*)-**BTX-21c7*anti***. Most hydrogen atoms are omitted for clarity. The optimization and ranking workflow are detailed in Section S1.7. Final geometry optimization: r^2scan-3c/SMD(CH_2Cl_2).

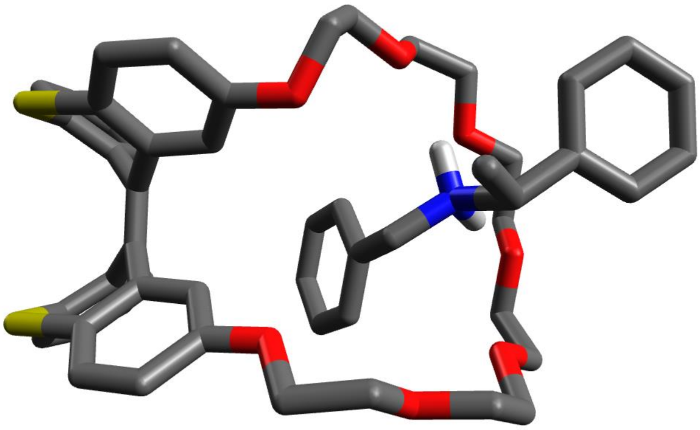

Figure S37. DFT-optimized structure for the lowest-energy conformer of (*R*)-**MDBA●BTX-21c7*syn***. Most hydrogen atoms are omitted for clarity. The optimization and ranking workflow are detailed in Section S1.7. Final geometry optimization: r^2scan-3c/SMD(CH_2Cl_2).

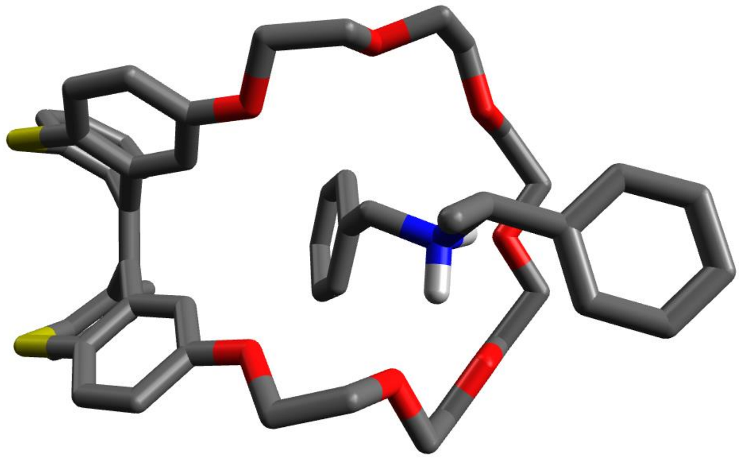

Figure S38. DFT-optimized structure for the lowest-energy conformer of (*S*)-**MDBA•BTX-21c7$_{syn}$**. Most hydrogen atoms are omitted for clarity. The optimization and ranking workflow are detailed in Section S1.7. Final geometry optimization: r^{2}scan-3c/SMD(CH_2Cl_2).

TD-DFT calculations at the ωB97X-D3/def2-TZVPP/CPCM(CH_2Cl_2) level of theory, without using the Tamm-Dancoff approximation were performed on some of the previously optimized lowest-energy conformers. The obtained results were in line with the observed experimental results, with similar CD spectra calculated for (*M*)-**BTX-21c7$_{anti}$** in the presence and after removal of a chiral **MDBA** guest and with a significant shift but preserved chirality of the axially-chiral dicationic (*M*)-**BTX-21c7^{2+}** (Figure S39).

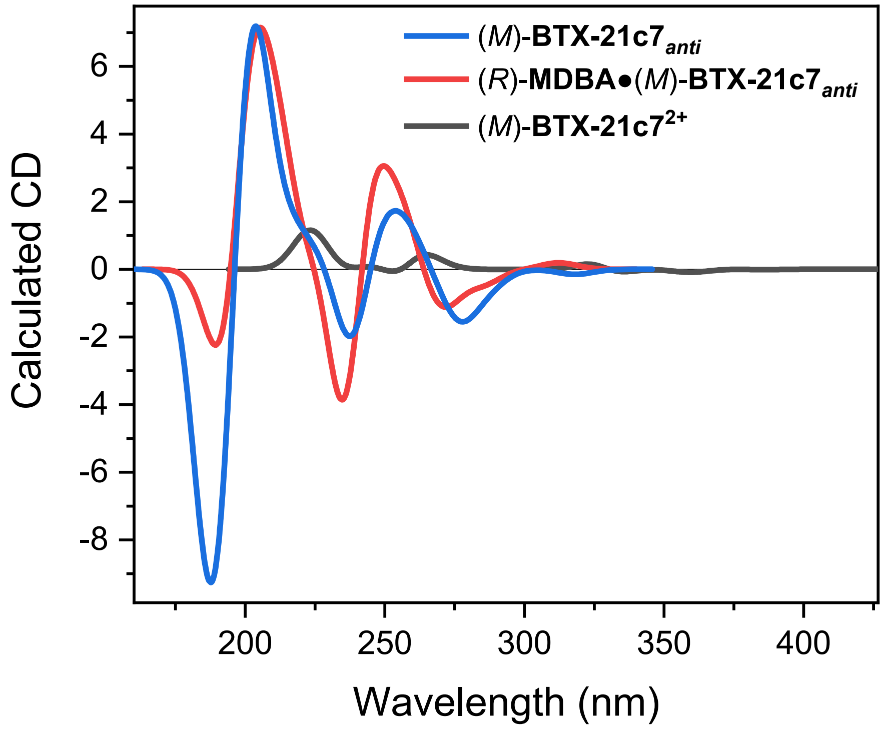

Figure S39. Calculated CD spectra for (*M*)-**BTX-21c7$_{anti}$**, (*R*)-**MDBA•**(*M*)-**BTX-21c7$_{anti}$** and (*M*)-**BTX-21c7^{2+}**. TD DFT, no TDA, ωB97X-D3/def2-TZVPP/CPCM(CH_2Cl_2)//r^{2}scan-3c/SMD(CH_2Cl_2). The optimization and ranking workflow are in Section 1.7.

The calculated CD spectrum for the host-guest complex (*R*)-**MDBA●**(*M*)-**BTX-21c7*anti*** was found to be in good agreement with the experimentally measured one for the chiral induction experiment involving (*R*)-**MDBA** and **BTX-21c7** after irradiation and relaxation to the (*R*)-**MDBA●**(*M*)-**BTX-21c7*anti*** enriched state (Figure S40), thus further confirming the assignment of isomers.

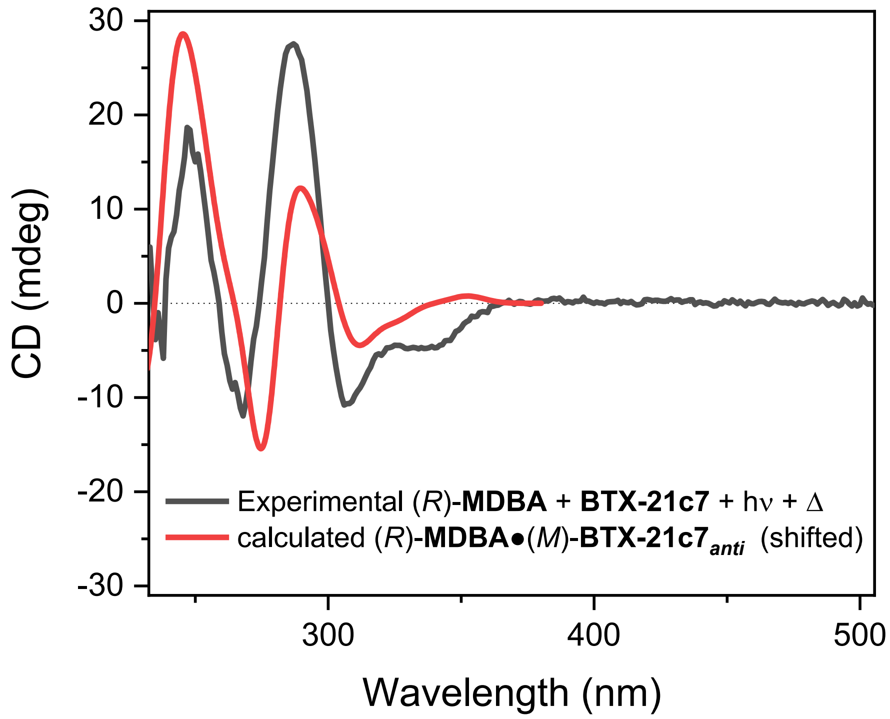

Figure S40. Red: Calculated CD spectra for (*R*)-**MDBA●**(*M*)-**BTX-21c7*anti***. The calculated intensity was multiplied by a factor 4 and the wavelength was shifted by +40 nm. TD DFT, no TDA, ωB97X-D3/def2-TZVPP/CPCM(CH_2Cl_2)//r^{2}scan-3c/SMD(CH_2Cl_2). The optimization and ranking workflow are detailed in Section 1.7. Black: experimental CD spectrum of 50 µM **BTX-21c7** in CH_2Cl_2 after chiral induction with (*R*)-**MDBA.**

The calculation procedure described in SI Section 1.7 also allowed for the determination of Boltzmann-averaged Gibbs free energies for every studied structure (*syn*- and *anti*-folded isomers of **BTX-21c7** the (*R*/*S*)-**MDBA** guest), see Table S1 and Table S2.

Table S1. Calculated Boltzmann-averaged Gibbs free energies differences (ΔG) between the free hosts: **BTX-21c7*anti*, BTX-21c7*syn***, and **BTX-21c7^{2+}**. Values are given in kJ/mol at 25 °C.

	**BTX-21c7**
**BTX-21c7$_{anti}$**	0
**BTX-21c7$_{syn}$**	33.7

Table S2. Calculated Boltzmann-averaged Gibbs free energies differences (ΔG) between the host-guest complexes of (*R/S*)-**MDBA** and **BTX-21c7**. Values are given in kJ/mol at 25 °C.

	(*R*)-**MDBA**	(*S*)-**MDBA**
**BTX-21c7*syn***	50.46	48.77
(*M*)-**BTX-21c7*anti***	0.16	4.11
(*P*)-**BTX-21c7*anti***	3.85	0

The calculated values indeed reproduce the stability trends that were determined experimentally: the binding of the point chiral guest to the (*P*/*M*) host yields diastereomers of different energies. A stronger binding of the (*R*) enantiomer of **MDBA** with the (*M*) enantiomer of **BTX-21c7*anti*** was obtained and oppositely the (*S*) enantiomer of **MDBA** displays a stronger binding with the (*P*) enantiomer of **BTX-21c7anti**. Nevertheless, the calculated binding energy differences $\Delta\Delta\mathrm{G} = \Delta\mathrm{G}_{R\bullet M} - \Delta\mathrm{G}_{R\bullet P}$ is overestimated, with an experimental value of −0.83 kJ/mol and a calculated value of −3.69 kJ/mol. Hence, these calculations efficiently qualitatively reproduced the overall behavior of the system which was observed experimentally.

These calculations also allow to rationalize the observed stability trends (and thus the favored helicity) from a structural point of view. In particular, we used the length of the four closest hydrogen bonding contacts (distance between N-donor and O-acceptors) as a structural indication of the quality of the fit of the guest into the host. These contacts are shorter for the more energetically-favored (*R*)-**MDBA●** (*M*)-**BTX-21c7** complex (2.946, 2.987, 3.071 and 3.275 Å) than for the diastereomeric (*S*)-**MDBA●**(*M*)-**BTX-21c7** complex (2.993, 3.008, 3.014 and 3.735 Å).

7. References

[1] Y. Liu, Q. Zhang, S. Crespi, S. Chen, X.-K. Zhang, T.-Y. Xu, C.-S. Ma, S.-W. Zhou, Z.-T. Shi, H. Tian, B. L. Feringa, D.-H. Qu, *Angew. Chem. Int. Ed.* **2021**, *60*, 16129-16138.

[2] S. Kuwahara, R. Chamura, S. Tsuchiya, M. Ikeda, Y. Habata, *Chem. Commun.* **2013**, *49*, 2186-2188.

[3] R. Hein, Y. Gisbert, B. L. Feringa, *J. Am. Chem. Soc.* **2025**, *147*, 13649-13657.

[4] P. Thordarson, *Chem. Soc. Rev.* **2011**, *40*, 1305-1323.

[5] S. Grimme, *J. Chem. Theory Comput.* **2019**, *15*, 2847-2862.

[6] P. Pracht, F. Bohle, S. Grimme, *Physical Chemistry Chemical Physics* **2020**, *22*, 7169-7192.

[7] P. Pracht, S. Grimme, C. Bannwarth, F. Bohle, S. Ehlert, G. Feldmann, J. Gorges, M. Müller, T. Neudecker, C. Plett, S. Spicher, P. Steinbach, P. A. Wesołowski, F. Zeller, *J. Chem. Phys.* **2024**, *160*.

[8] C. Bannwarth, S. Ehlert, S. Grimme, *J. Chem. Theory Comput.* **2019**, *15*, 1652-1671.

[9] S. Grimme, F. Bohle, A. Hansen, P. Pracht, S. Spicher, M. Stahn, *J. Phys. Chem. A* **2021**, *125*, 4039-4054.

[10] F. Neese, F. Wennmohs, U. Becker, C. Riplinger, *J. Chem. Phys.* **2020**, *152*.

[11] S. Grimme, A. Hansen, S. Ehlert, J.-M. Mewes, *J. Chem. Phys.* **2021**, *154*.

[12] A. V. Marenich, C. J. Cramer, D. G. Truhlar, *J. Phys. Chem. B* **2009**, *113*, 6378-6396.

[13] V. Barone, M. Cossi, *J. Phys. Chem. A* **1998**, *102*, 1995-2001.

[14] S. Grimme, J. Antony, S. Ehrlich, H. Krieg, *J. Chem. Phys.* **2010**, *132*.

[15] Y.-S. Lin, G.-D. Li, S.-P. Mao, J.-D. Chai, *J. Chem. Theory Comput.* **2013**, *9*, 263-272.

[16] F. Weigend, R. Ahlrichs, *Phys. Chem. Chem. Phys.* **2005**, *7*, 3297-3305.

[17] S. Dasgupta, J. Wu, *Chem. Sci.* **2012**, *3*, 425-432.
